# Novel Mutations Associated With Various Types of Corneal Dystrophies in a Han Chinese Population

**DOI:** 10.3389/fgene.2019.00881

**Published:** 2019-08-29

**Authors:** Jing Zhang, Dan Wu, Yue Li, Yidan Fan, Huiyu Chen, Jiaxu Hong, Jianjiang Xu

**Affiliations:** ^1^Department of Ophthalmology and Visual Science, Eye Institute, Eye & ENT Hospital, Shanghai Medical College of Fudan University, Shanghai, China; ^2^NHC Key Laboratory of Myopia, Fudan University, Shanghai, China; ^3^Shanghai Key Laboratory of Visual Impairment and Restoration, Shanghai, China

**Keywords:** corneal dystrophies, next-generation sequencing, targeted-region sequencing, Han Chinese population, mutations

## Abstract

**Aims:** To study the genetic spectra of corneal dystrophies (CDs) in Han Chinese patients using next-generation sequencing (NGS).

**Methods:** NGS-based targeted region sequencing was performed to evaluate 71 CD patients of Han Chinese ethnicity. A custom-made capture panel was designed to capture all coding exons and untranslated regions plus 25 bp of intronic flanking sequences of 801 candidate genes for eye diseases. The Genome Analysis Tool Kit Best Practices pipeline and an intensive computational prediction pipeline were applied for the analysis of pathogenic variants.

**Results:** We achieved a mutation detection rate of 59.2% by NGS. Eighteen known mutations in CD-related genes were found in 42 out of 71 patients, and these cases showed a genotype–phenotype correlation consistent with previous reports. Nine novel variants that were likely pathogenic were found in various genes, including CHST6, TGFBI, SLC4A11, AGBL1, and COL17A1. These variants were all predicted to be protein-damaging by an intensive computational analysis.

**Conclusions:** This study expands the spectra of genetic mutations associated with various types of CDs in the Chinese population and highlights the clinical utility of targeted NGS for genetically heterogeneous CD.

## Introduction

Corneal dystrophies (CDs) are a group of inherited disorders affecting the cornea, many of which may lead to progressive visual impairment through corneal opacification caused by decompensation, deposition, or scarring (Bron, 1990). Anatomically, CDs can be divided into epithelial and subepithelial dystrophies, epithelial–stromal TGFBI dystrophies, stromal dystrophies, and endothelial dystrophies (Weiss et al., 2015). The overall prevalence of CDs is estimated to be 0.13% in the general population (Musch et al., 2011), but the prevalence of each individual type may vary among racial and ethnic groups (Smith and Taylor, 1991; Jonasson et al., 1996; Fujiki et al., 2001; Gwilliam et al., 2005). Corneal transplantation is currently the most effective treatment of CDs, although there is a potential risk for the donated cornea to be affected by the disease (Marcon et al., 2003; Cheng et al., 2013), highlighting the urgent need for intensively deciphering the etiology of CDs.

Genetic factors are considered to play predominant roles in the pathogenesis of CDs. With a few notable exceptions, CDs are usually Mendelian disorders and exhibit an autosomal dominant pattern of inheritance (Aldave, 2011; Vincent, 2014). To date, disease-causing mutations in 18 genes have been identified, and many CDs have been genetically characterized (Weiss et al., 2015). For example, direct correlations have been established between mutations in CHST6, UBIAD1, SLC4A11, PIKFYVE, TACSTD2, and DCN with macular corneal dystrophies (MCD) (Akama et al., 2000), Schnyder CD (Nowinska et al., 2014a), congenital hereditary endothelial dystrophy (Vithana et al., 2006), fleck CD (Li et al., 2005), gelatinous drop-like CD (Tsujikawa et al., 1999), and congenital stromal CD (Bredrup et al., 2010), respectively. Interestingly, significant genetic heterogeneity also exists. For instance, different mutations in TGFBI have been recognized to cause various types of CDs, including granular corneal dystrophies (GCDs), lattice CD type 1, Reis–Bucklers CD, and Thiel–Benke CD, as well as some other atypical CDs (Han et al., 2016). Mutations in KRT3 (Szaflik et al., 2008) and KRT12 (Corden et al., 2000) are both responsible for Meesmann CD, with no distinguishable difference in clinical appearance and disease course. Similarly, variants in ZEB1 (Lechner et al., 2013), COL8A2 (Kuot et al., 2014), TCF4 (Baratz et al., 2010), FEN1 (Wojcik et al., 2014), AGBL1 (Riazuddin et al., 2013), and LOXHD1 (Riazuddin et al., 2012) all cause Fuchs endothelial corneal dystrophy (FECD), while the age of onset may vary. This wide range of heterogeneity can render the genetic diagnosis of CDs challenging.

Targeted region sequencing (TRS) has an excellent performance for the molecular diagnosis of genetically heterogeneous disorders (Taylor et al., 2017). By simultaneously sequencing several hundreds of potential disease-associated genes, TRS provides a comprehensive overview of genetic profiles linked to the affected phenotypes. In addition, the higher coverage, lower cost, and relative ease of data interpretation have made this approach more common in routine clinical diagnostic settings than whole-exome sequencing and whole-genome sequencing. In this study, we applied a TRS approach covering all regions of interest in 801 candidate genes for eye diseases to examine the genetic spectra of CDs in a cohort of 71 Han Chinese patients. Eighteen known CD-causing mutations were found in 42 out of 71 patients, indicating a mutation detection rate of 59.2% using TRS. In addition, we identified nine novel variants that were likely pathogenic in 10 patients. These novel variants were all predicted to be protein-damaging by an intensive computational analysis. The current study expands the spectra of genetic mutations associated with various types of CDs in the Han Chinese population and indicates that TRS is an efficient approach for the detection of mutations underlying CDs with potential significance in clinical practice.

## Materials and Methods

### Subjects

Seventy-one patients with various types of CDs, including 54 sporadic cases and 17 cases from 10 unrelated families, were recruited from the ophthalmology clinic at the Eye and ENT Hospital of Fudan University from October 2015 to May 2018. Ophthalmological examinations included visual acuity, refractive measurement, intraocular pressure, slit-lamp examination, and in some cases, typical in vivo confocal microscopy (IVCM) examination (Le et al., 2017). Inclusion in the CD diagnostic group was based on the following criteria: 1) Clinical diagnosis of epithelial–stromal dystrophies depended on the observation of refractile lattice lines (lattice CD type 1), discrete gray-white deposits (GCD1), both granular, stellate opacities and lattice lesions (GCDII), or irregular gray-white haze in the subepithelium and stroma of the cornea (atypical). 2) Clinical diagnosis of MCD was based on the slit-lamp findings: ground glass-like haze in the superficial stroma and multiple small, gray-white opacities with irregular borders within this hazy matrix. 3) The diagnosis of endothelial CD was based on the observation of corneal clouding ranging from diffuse haze to ground glass milky appearance with occasional focal gray spots, as well as the disease onset age.

The CDs were initially classified according to their phenotypic appearance and anatomic locations, and a more precise diagnosis was received combining genetic testing result, as described by the International Committee for Classification of Corneal Dystrophies, Second Edition (Weiss et al., 2015). Patients with extraocular somatic defects or other ocular or developmental abnormalities were excluded from this study. All participants provided written informed consent. There was no known consanguinity in any of these families. The whole research procedure was performed in accordance with the Declaration of Helsinki and was approved by the Ethics Committee of the Eye and ENT Hospital.

Genomic DNA was isolated from the peripheral blood using the QIAGEN FlexiGene DNA Kit (Qiagen, Hamburg, Germany) according to the manufacturer’s instructions. A total of 100 ethnically matched subjects without a history of ocular diseases were recruited as healthy controls.

### Library Preparation and Targeted Region Sequencing

A custom-made capture panel was designed to capture all coding exons and untranslated regions (UTRs) plus 25 bp of intronic flanking sequences of 801 candidate genes for eye diseases. All introns of TCF4 were also included in the capture panel. This panel included 18 known causal genes for CDs retrieved from the Online Mendelian Inheritance in Man database and the Human Gene Mutation Database (TGFBI UBIAD1 CHST6 VSX1 PIKFYVE DCN KRT12 KRT3 ZEB1 SLC4A11 COL8A2 COL17A1 TCF4 FEN1 AGBL1 LOXHD1 TACSTD2 and GSN), 495 eye disease-causing genes obtained from NEIBank (http://neibank.nei.nih.gov/), and other potentially associated genes collected from the literature (see **Table S1** for a full list of examined genes). The genomic DNA of the probands was sonicated to obtain fragments of 200–300 bp. Library capture was performed using the custom NimbleGene SeqCap EZ Choice Enrichment Kit (Roche, Basel, Switzerland) and sequenced using an Illumina HiSeq2500 Analyzer (San Diego, CA) with a 2 × 150-bp paired-end read protocol (Biomarker Technologies, Beijing, China). Image analysis and base calling were conducted using the Illumina Pipeline to generate raw data.

### Variant Identification and Validation

Clean reads that passed quality control were aligned to the human genome reference from the National Center for Biotechnology Information (NCBI) database (GRCh37/hg19) using the Burrows Wheeler Aligner package. Single nucleotide variants and insertions and deletions were called using the Genome Analysis Tool Kit and annotated using the ANNOVAR package. Variants with a coverage of less than 10× were removed. Variants were then checked for their minor allele frequencies (MAFs) in dbSNP144, 1000 Genome Project phase 3, Esp6500, ExAC, and gnomAD databases, and those with MAFs in East Asians ≥5% were filtered out. Variants were evaluated for possible pathogenic clinical significance according to the 2015 American College of Medical Genetics and Genomics (ACMG) guidelines (Richards et al., 2015) based on a combination of published studies and computational, functional, and population data. The amino acid sequences of candidate genes from several species were retrieved from NCBI GenBank, and conservation was evaluated using Clustal Omega. Variants that were classified as pathogenic or likely pathogenic according to the ACMG guidelines were validated by Sanger sequencing (see **Table S2** for primer sequences).

### Prediction of the Effects of Novel Missense Mutations on Protein Stability, Function, and Physiochemical Properties

Bioinformatics tools were applied to investigate a range of physicochemical effects of each missense mutation on the structure and function of the corresponding protein. The following tools were used to evaluate the change in protein stability: I-Mutant 3.0 (http://gpcr.biocomp.unibo.it/cgi/predictors/I-Mutant3.0/I-Mutant3.0.cgi), ProSMS (http://babel.ucmp.umu.se/prosms/), and MUpro (http://mupro.proteomics.ics.uci.edu/). The tools used to predict the pathogenicity of missense mutations were PredictSNP (https://loschmidt.chemi.muni.cz/predictsnp/), MAPP (http://mendel.stanford.edu/SidowLab/downloads/MAPP/), and PhD-SNP (http://snps.biofold.org/phd-snp/phd-snp.html). Changes in the physiochemical properties of the native and variant amino acids were predicted using NCBI Amino Acid Explorer.

### Homology Modeling

The crystal structure of the human TGFBI fasciclin (FAS) 1–4 domain was obtained from Protein Data Bank (PDB 2LTB). The native molecule at position 565 was individually mutated from leucine to histidine or proline using the SwissPDB viewer. Homology modeling on the wild-type or the mutant protein was performed using the SWISS-MODEL server (https://swissmodel.expasy.org/). Taking the resolved structure of human Band 3 anion transport protein at 3.5-Å resolution (PDB 4YZ, amino acid identity = 32.78%) as a template, the model structure of wild-type SLC4A11 was created by Swiss-Model Server. The three-dimensional (3D) homology model of human AGBL1 was built with the crystal structure of human putative carboxypeptidase at 2.5-Å resolution (PDB 3K2K, amino acid identity = 24.60%) as a template. For CHST6 and COL17A1, the homology models generated by the Swiss-Model Server do not cover the positions of the mutations. Thus, the 3D homology models of these two proteins were created using I-TASSER (https://zhanglab.ccmb.med.umich.edu/I-TASSER). Structures of the wild-type and mutant proteins were visualized using PyMol (http://www.pymol.com).

## Results

### Targeted Region Sequencing Analysis

We performed TRS for 71 patients (31 males and 40 females) referred with clinical indications of various CDs (**Table 1**). The average age of these patients was 45.1 ± 16.3 years. An average of 5,562,101 aligned sequencing reads were generated for each patient. The mean depth of coverage in the targeted regions was 231× across all samples, and 99.3% of amplicon regions were covered at ≥10×. An average of 2,791 variants in targeted regions were obtained for each sample, and after variants with low coverage (<10×) and common variants that existed in public databases (MAF ≥ 5%) were removed; the average number of variants in each sample was reduced to 332. The average ratios of homozygous variants to heterozygous variants in targeted exons and UTRs were 1:18.4 and 1:10.0, respectively. These variants were further filtered according to the ACMG guidelines and validated by Sanger sequencing. Overall, 18 known CD-causing mutations were found in 42 out of 71 patients, indicating a mutation detection rate of 59.2% using NGS. In addition, we identified nine novel likely pathogenic variants in 10 patients (two additional cases harbored a novel mutation and a known mutation simultaneously). CHST6 and TGFBI variants were most frequently identified, together accounting for 45/71 cases (63.4%). In addition, we detected pathogenic or likely pathogenic variants in SLC4A11 in four cases, AGBL1 in two cases, and COL17A1 in one case (**Table 2**). Nineteen cases had no variants of interest.

**Table 1:** Clinical information for included cases with various types of CD.

	Number of cases	Average age (mean ± SD)	Male cases (%)
***CD cases with defined mutations***			
Macular corneal dystrophy, stromal	23	40.4 ± 11.8	34.8
Epithelial-stromal CD	22	50.0 ± 15.6	36.4
Endothelial CD, congenital	4	19.2 ± 13.8	100
Fuchs endothelial CD	3	47.7 ± 3.5	50
***CD cases with unknown mutations***			
CD, stromal	5	51.6 ± 18.4	40
Endothelial CD, congenital	1	1.5	100
Fuchs endothelial CD	13	53.6 ± 11.6	38.5

**Table 2:** Pathogenic or likely pathogenic variants identified in targeted region sequencing.

Gene Refseq ID	Predicted AA change	Affected cases	*In silico* prediction:	MAF in ExAc,1KG	Zygosity
CHST6 NM_021615	p.S51X	1	D,D,D	0,0	hom
	**p.F55S**	1	D,D,D	0,0	het
	**p.F55S**, p.Y358H	2	D,D,D	0,0	com-Het
	**p.P133R**	2	D,D,D	0,0	het
	p.C149G	1	D,D,D	0,0	hom
	**p.C165X**	1	D,D,D	0,0	het
	p.R177C	1	D,D,D	0,0	hom
	p.D203N	2	D,D,T	0,0	hom
	p.R205W	1	D,D,D	0.002,0	het
	p.R211G	3	D,D,D	0,0	het
	p.R205W, p.R211G	2			com-Het
	p.R211G,p.E254X	1	D,D,D	0,0	com-Het
	p.R211Q	1	D,D,D	0,0	hom
	p.W232X	2	D,D,D	0,0	hom
	p.E254X	1	D,D,D	0,0	hom
	p.Y358H	1	D,D,D	0,0	hom
TGFBI NM_000358	p.R124C	5	D,D,D	0,0	het
	p.R124L	3	D,D,D	0,0	het
	p.R124H	8	D,D,D	0.007,0	het
	p.F540S	2	D,D,D	0,0	het
	p.R555W	2	D,D,D	0,0	het
	**p.L565H**	2	D,D,D	0,0	het
SLC4A11 NM_032034	**p.G413R, p.L732fs**	1	D,D,D	0,0	com-Het
	**p.Q676R**	1	D,D,D	0,0	hom
	p.R755W	1	D,D,D	0,0	hom
	p.R869H	1	D,D,D	0,0	hom
AGBL1 NM_152336	**p.R748H**	1	D,D,D	0.0037,0.002	het
	p.R1028X	1	D,D,D	0,0	het
COL17A1 NM_000494	**p.P1185L**	1	D,D,D	0,0	hom

MAF, minor allele frequency; ExAc, the exome aggregation consortium; 1KG, 1,000 genome projects; D, damaging; T, tolerant; Hom, homozygous; Het, heterozygous; com-Het, compound heterozygous. In silico prediction was performed by SIFT, PolyPhen2, and Mutation Taster; Novel variants identified in the current study were shown in bold font.

### Novel Missense, Nonsense Mutations in Key Domains of CHST6

**Figure 1:**
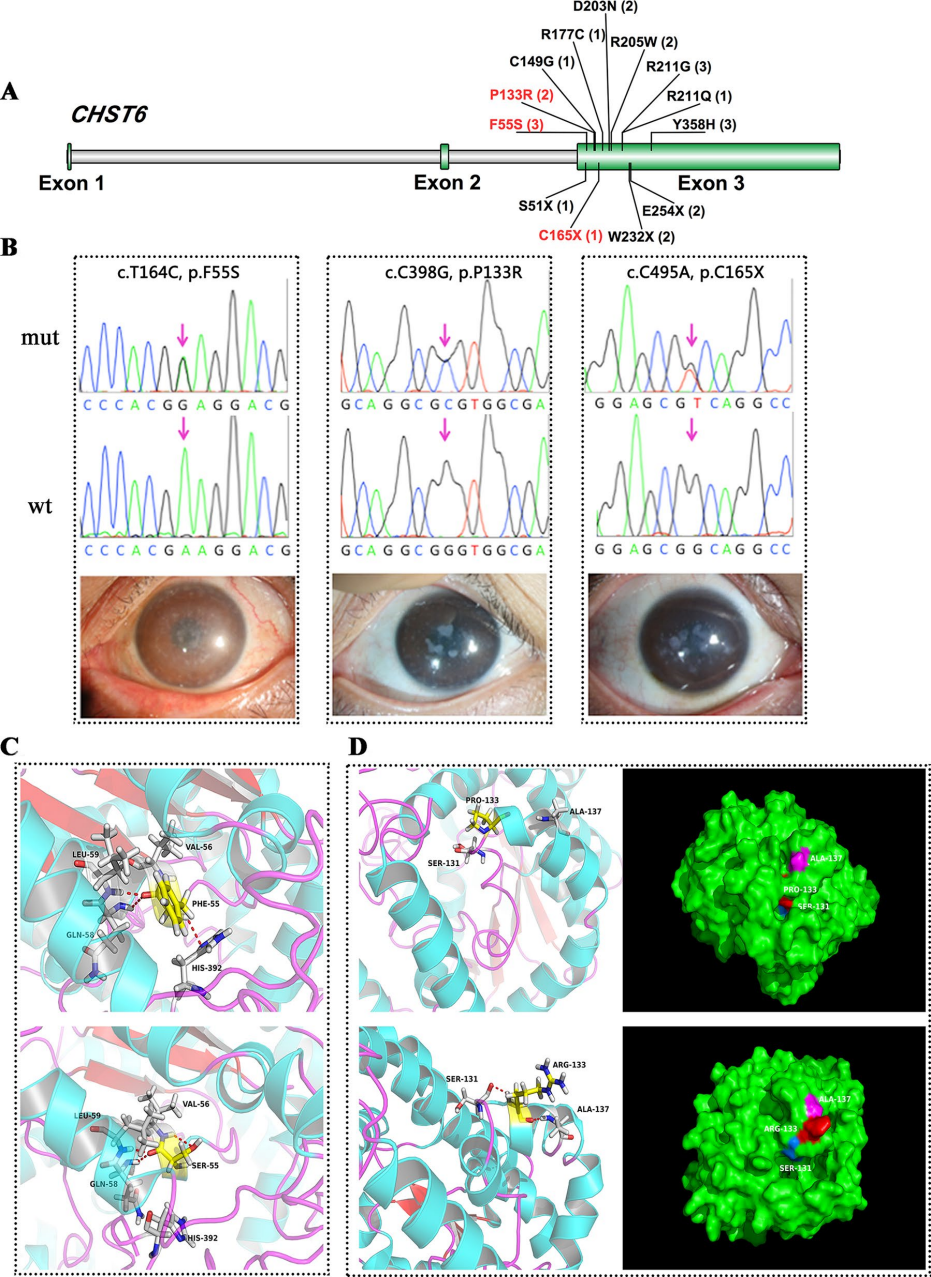
CHST6 mutations identified in this study. **(A)** Schematic diagram of CHST6 exons. The positions of mutations found in this study were labeled, and novel variants were indicated in red. Number of affected individuals was shown in brackets. **(B)** Validation of the three novel CHST6 mutations by Sanger sequencing, and representative images of the patients that harbored the novel mutations. **(C)** 3D homology model changes induced by the p.F55S mutation. Hydrogen bonds were shown as red dashed lines. **(D)** 3D homology model and surface changes induced by the p.P133R mutation.

CHST6 encodes an enzyme that mediates the sulfation of keratan in the cornea, which functions in the maintenance of corneal transparency. Defects in CHST6 are known to cause MCD. Twenty-three patients had likely causative mutations in the CHST6 gene (23/71, 32.4%), including 4 familial cases and 19 sporadic cases. Many of these variants have been reported before, and these patients had a genotype–phenotype correlation consistent with prior reports. Three novel mutations were identified in this study, including two missense mutations and one stop-gain mutation (**Figure 1A**).

We found a novel heterozygous CHST6 missense mutation (c.T164C, p.F55S) in three cases, two of which also harbored a previously validated mutation, p.Y358H in CHST6 (**Figure 1B**, left). A shared novel missense mutation (c.C398G, p.P133R) was found in two siblings from one affected family (**Figure 1B**, middle). We also observed a novel nonsense mutation (c.C495A, p.C165X) in another male case (Figrue 1B, right). All three novel variants were absent in 100 in-house healthy controls and were predicted to be protein damaging by an in silico analysis (**Table 2**).

### TGFBI Corneal Dystrophies

**Figure 2:**
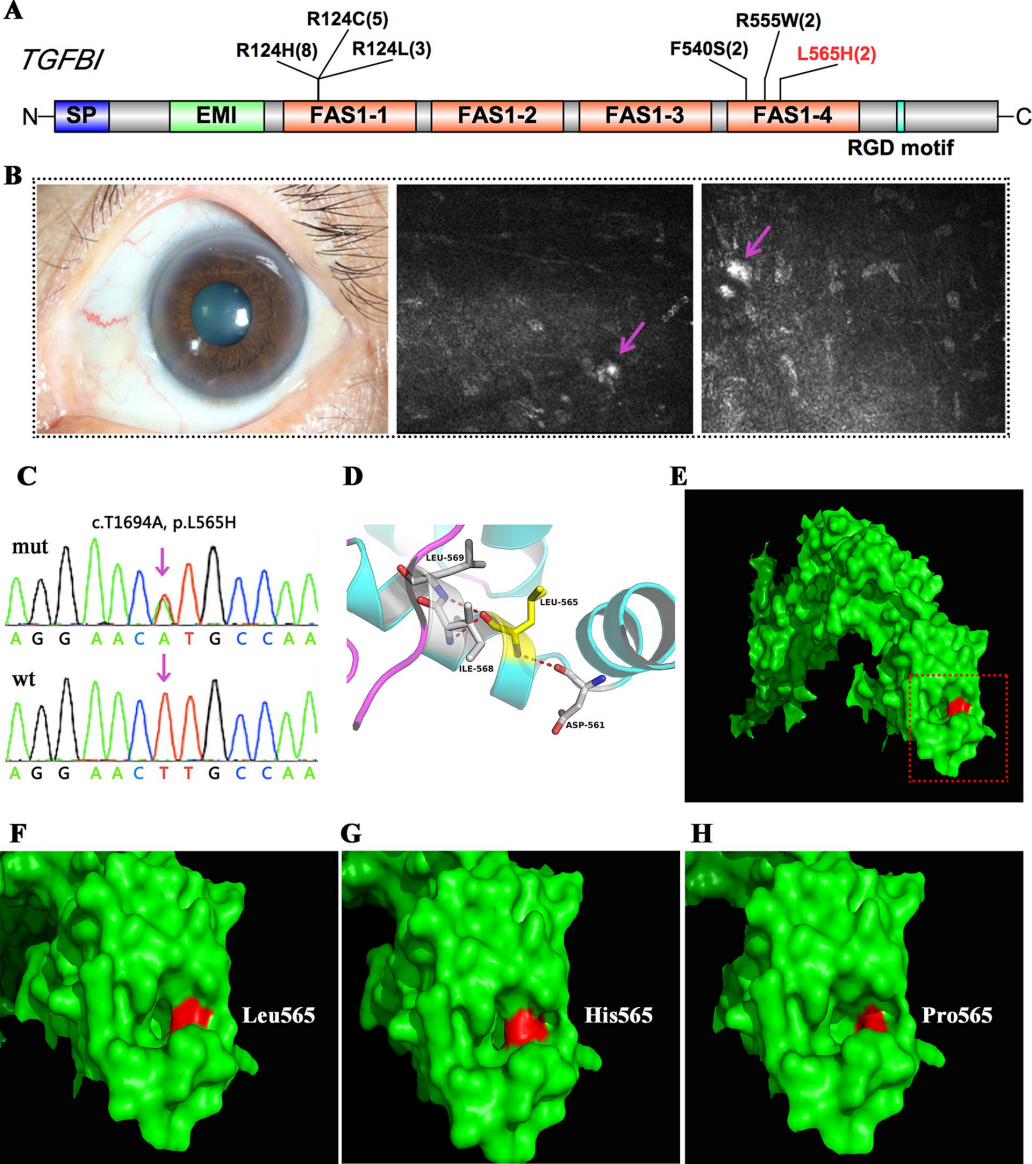
TGFBI mutations identified in this study. **(A)** Schematic diagram of TGFBI protein structure including the four fascilin-like (FAS1 1-4) domains. **(B)** Representative image and *in vivo* confocal microscopy (IVCM) examination of the patient that harbored the p.L565H mutation. **(C)** Validation of the p.L565H mutations by Sanger sequencing. **(D)** 3D homology model of the TGFBI protein (partial). **(E–H)** Surface changes caused by the p.L565H or p.L565P mutation. The mutations affected the size of the nearby cavity. **F–H** is a magnification of the area shown in red rectangle in **E**.

TGFBI encodes transforming growth factor beta-induced protein (TGFBIp), a 68-kDa extracellular matrix protein composed of a secretory signal peptide, a cysteine-rich EMI domain, four homologous FAS1 domains, and an arginine–glycine–aspartate motif that binds to integrin at the C-terminus. TGFBIp plays a role in cell–collagen interactions, and the accumulation of insoluble protein deposits is responsible for many different types of CDs. In this study, mutations in TGFBI were found in 22 patients, and the majority of these patients (18/22) harbored mutations in one of two hot spots at codon Arg124 in the first FAS1 domain or Arg555 in the fourth FAS1 domain. Two affected members in one family had a shared heterozygous F540S mutation, also located in the fourth FAS1 domain (**Table 2** and **Figure 2A**). All of these patients had a genotype–phenotype correlation consistent with prior reports. Interestingly, we found a novel heterozygous p.L565H mutation in TGFBI in two unrelated cases (**Figure 2C, D**). A slit-lamp examination revealed slightly diffuse opacities and haze in the central cornea of the patient. IVCM showed oval or short comma-shaped highlighted spots, which were mainly localized in the stroma, leading to diffuse clouding. No typical lattice lines were present (**Figure 2B**).

### SLC4A11 Mutations Associated With Corneal Endothelial Dystrophy

**Figure 3:**
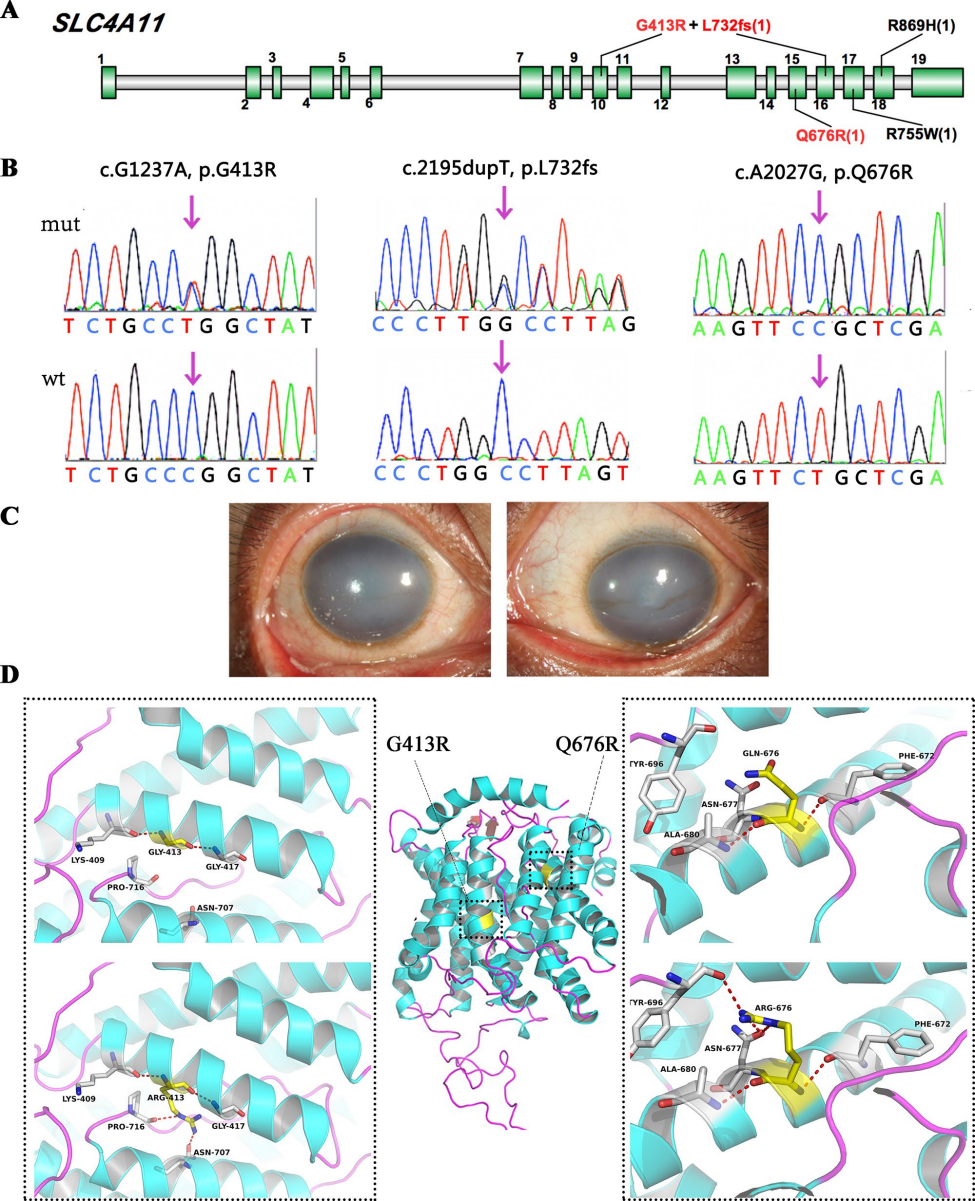
SLC4A11 mutations identified in this study. **(A)** Schematic diagram of SLC4A11 exons. **(B)** Validation of the three novel SLC4A11 mutations by Sanger sequencing. **(C)** Representative images of the patient that harbored double heterozygous mutations, p.G413R and p.L732fs. **(D)** 3D homology model changes induced by the missense mutations. Hydrogen bonds were shown as red dashed line.

SLC4A11 encodes a membrane transport protein (OH-/H+/NH3/H2O) of the basolateral corneal endothelium; mutations in this gene cause some cases of congenital hereditary endothelial dystrophy and FECD. In this study, we detected five SLC4A11 variants in four cases, and three of these variants were novel (**Figure 3A**).

We found two novel heterozygous variants in SLC4A11 in one male case (**Figure 3C, D**), including a frameshift variant at position 732, and a missense mutation, p.G413R. They were compound heterozygous mutations, as determined by parental genotype (**Figure S1**). A novel homozygous SLC4A11 p.Q676R mutation was identified in another male. These missense mutations were not observed in normal populations, and they were predicted to be damaging based on an in silico analysis (**Figure 3B** and **Table 2**). In addition, two cases carried previously validated SLC4A11 mutations, p.R755W and p.R869H, and their phenotypes were consistent with those described in previous reports.

### Atypical Corneal Dystrophies Caused by Mutations in AGBL1 and COL17A1

**Figure 4:**
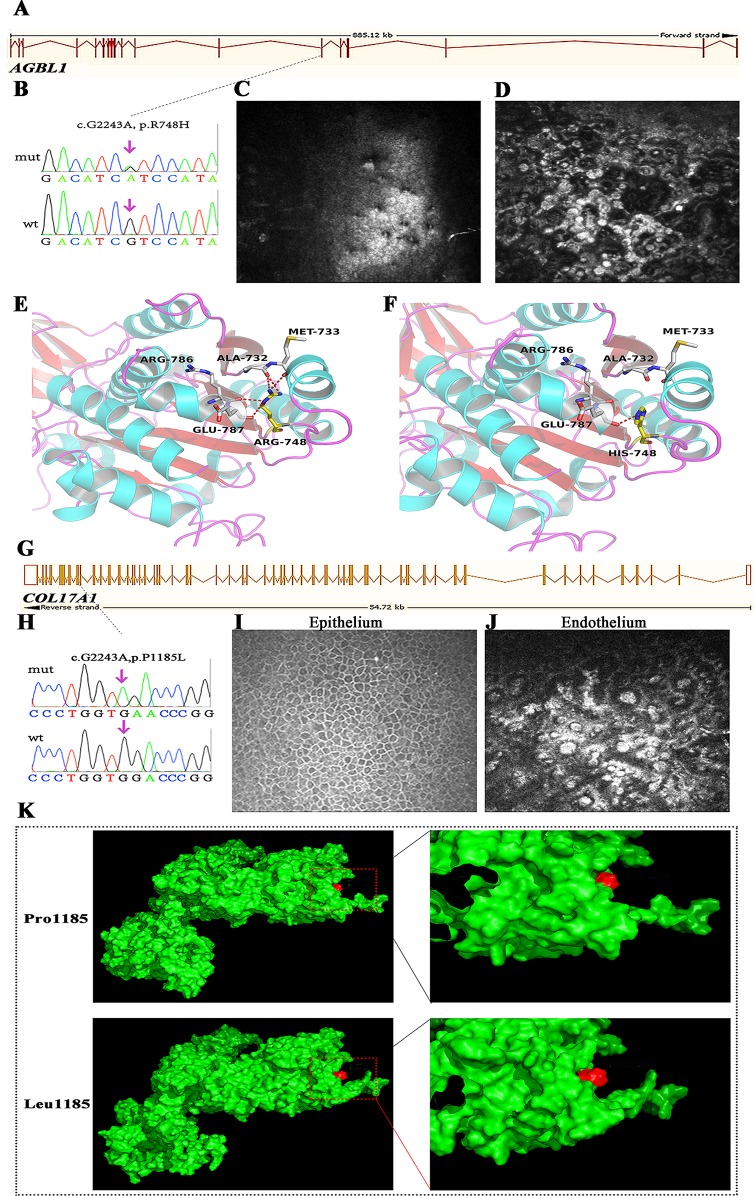
Mutations in AGBL1 **(A–F)** and COL17A1 **(G–K)** that identified in this study. **(A)** Schematic diagram of AGBL1 exons. The position of the p.R748H mutation was labeled with dashed line. **(B)** Validation of the AGBL1 p.R748H mutation by Sanger sequencing. **(C, D)** *In vivo* confocal microscopy (IVCM) examination of the patient that harbored the p.R748H mutation, shown as the lesions in corneal endothelium. **(E, F)** 3D homology model changes induced by the AGBL1 p.R748H mutation. **(G)** Schematic diagram of COL17A1 exons. The position of the p.P1185L mutation was labeled with dashed line. **(H)** Validation of the COL17A1 p.P1185L mutation by Sanger sequencing. **(I, J)** IVCM examination of the patient that harbored the p.P1185L mutation, shown as the normal corneal epithelium **(I)** and lesions in corneal endothelium **(J)**. **(K)** Surface changes caused by the p.P1185L mutation. The mutation showed blocked entrance of the adjacent cavity. The residue in position 1185 was labeled in red.

AGBL1 encodes a glutamate decarboxylase that catalyzes the deglutamylation of polyglutamylated proteins. Mutations in this gene cause dominant late-onset Fuchs corneal dystrophy. In this study, we found AGBL1 mutations in two cases, including the previously validated truncated mutation p.R1028X and the novel missense mutation p.R748H (**Figures 4A, B**). IVCM examination showed pleomorphism and hyporeflective structures, surrounded by guttae in corneal endothelial cells, consistent with the characteristics of FECD (**Figures 4C, D**). Interestingly, both patients showed disease symptoms, such as pain, photophobia, and visual impairment, at around 40 years old. The age of disease onset for these two patients was earlier than that of traditional late-onset FECD, which usually begins in the fifth or sixth decade of life.

We found a 51-year-old male with severe visual recession staring from the fourth decade of life. Although he felt no pain, his visual acuity by hand movement was 10 cm in the right eye and 0.02 cm in the left eye, resulting from a corneal central opacity. IVCM examination showed the formation of guttae and microscopic protrusions of the basal lamina underlying his corneal endothelium (**Figure 4J**), characteristic of FECD. However, genetic screening of known FECD-associated genes (including ZEB1, COL8A2, TCF4, FEN1, AGBL1, and LOXHD1) showed no pathogenic variants. Interestingly, we found a novel homozygous mutation, p.P1185L, in COL17A1 in this patient (**Figures 4G, H**). Mutations in COL17A1 cause epithelial recurrent erosion dystrophy; however, the corneal epithelium of this patient was normal (**Figure 4I**).

### *In Silico* Analysis of Novel Missense Mutations That Are Likely Pathogenic

We used several in silico bioinformatics tools to predict the pathogenicity and stability of the seven newly identified missense mutations. As shown in **Table 3**, these mutations were predicted to be pathogenic and destabilizing by most of these tools, further supporting their contributions to disease pathogenesis. In addition, many of these mutations influenced the charge, polarity, hydrophobicity, or side chain flexibility of the corresponding proteins. Furthermore, for the seven novel missense mutations, we examined the 3D homology structures of their corresponding proteins; we found that all of the mutations altered the atomic environment surrounding the substitution site. For example, as shown in **Figure 1C**, Phe-55 in CHST6 stabilized a nearby loop through a hydrogen bond with His-392, while the substitution with a polar and hydrophilic Serine abolished this interaction, altering the local environment. For the CHST6 p.P133R mutation, although new contacts with the adjacent amino acids Ser-131 and Ala-137 were introduced, the p.P133R exchange generated more pronounced changes in shape and charge distribution at the site (**Figure 1D**). Such surface changes were also induced by mutations in TGFBI and COL17A1. The TGFBI L565H and L565P substitutions resulted in conformational changes in neighboring amino acids (**Figures 2E–H**) and affected the size of the nearby cavity. Similarly, the change from Pro to Leu at position 1,185 in COL17A1 also blocked the entrance of the adjacent cavity (**Figure 4K**). For mutations in SLC4A11, substitutions with a bulky, positively charged arginine at positions 413 and 676 altered the local charge environment, and interactions with the nearby helix and loop were also introduced (**Figure 3D**). The replacement from Arg to His at position 748 in AGBL1 did not alter physiochemical properties; however, the contacts with the adjacent helix and beta sheet were nearly abolished by this mutation (**Figures 4E, F**).

**Table 3:** Pathogenicity, stability, and changes in the physiochemical properties (PP) of the missense variants identified in this study.

Tools	Mutations	*CHST6*		*TGFBI*	*SLC4A11*		*AGBL1*	*COL17A1*
		p.F55S	p.P133R	p.L565H	p.G413R	p.Q676R	p.R748H	p.P1185L
Stability Prediction	I-mutant2	DE	DE	DE	DE	DE	DE	DE
	ProSMS	DS	N	DS	N	DS	N	N
	INPS-MD	DE	DE	DE	DE	DE	DE	DE
	Mupro	DE	IN	DE	IN	DE	DE	DE
Pathogenicity prediction	PredictSNP	PA	PA	PA	PA	PA	PA	N
	MAPP	PA	PA	PA	PA	PA	PA	N
	PhD-SNP	PA	PA	PA	PA	PA	PA	N
	SNAP	PA	PA	P	PA	P	N	PA
Changes in PP	Charge	Both N	N - P+	N - P+	N - P+	N - P+	Both P+	Both N
	Polarity	NPL-PL	NPL-PL	NPL-PL	NPL-PL	NPL-PL	PL	NPL
	HO/HI	HO-HI	HO-HI	HO-HI	HO-HI	HO-HI	Both HI	Both HO
	Side Chain flexibility	M-L	R-H	Both M	None- H	Both H	H-M	R-M

DE, decrease; IN, increase; DS: destabilizing; N, neutral; PA, pathogenic; P+, positive; PL, polar; NPL, nonpolar; HO, hydrophobicity; HI, hydrophilicity M, moderate; L, low; R, restricted; H, high.

## Discussion

We obtained a mutation detection rate of near 60% in 71 Han Chinese sporadic and familial cases referred with various types of CDs. In addition, we found nine novel variants that were likely pathogenic. These novel variants were all predicted to be protein-damaging by an intensive computational analysis. In general, most CD patients had a genotype-phenotype correlation consistent with prior reports, that is, we found CHST6 mutations in 23/28 stromal CD patients with macular appearance, TGFBI mutations in all the 22 patients with epithelial–stromal CD, as well as SLC4A11 mutations in 4/5 patients with congenital endothelial CD. However, we were unable to find a clear genotype–phenotype correlation in patients with FECD, as 13 FECD patients had unclear genetic etiology, and three patients showed atypical CDs caused by mutations in AGBL1 and a novel suspected causal gene, COL17A1. The current study expands the spectra of genetic mutations that are associated with various types of CDs in a Chinese population and highlights the clinical utility of TRS in the diagnostic setting of genetically heterogeneous CD.

**Figure 5:**
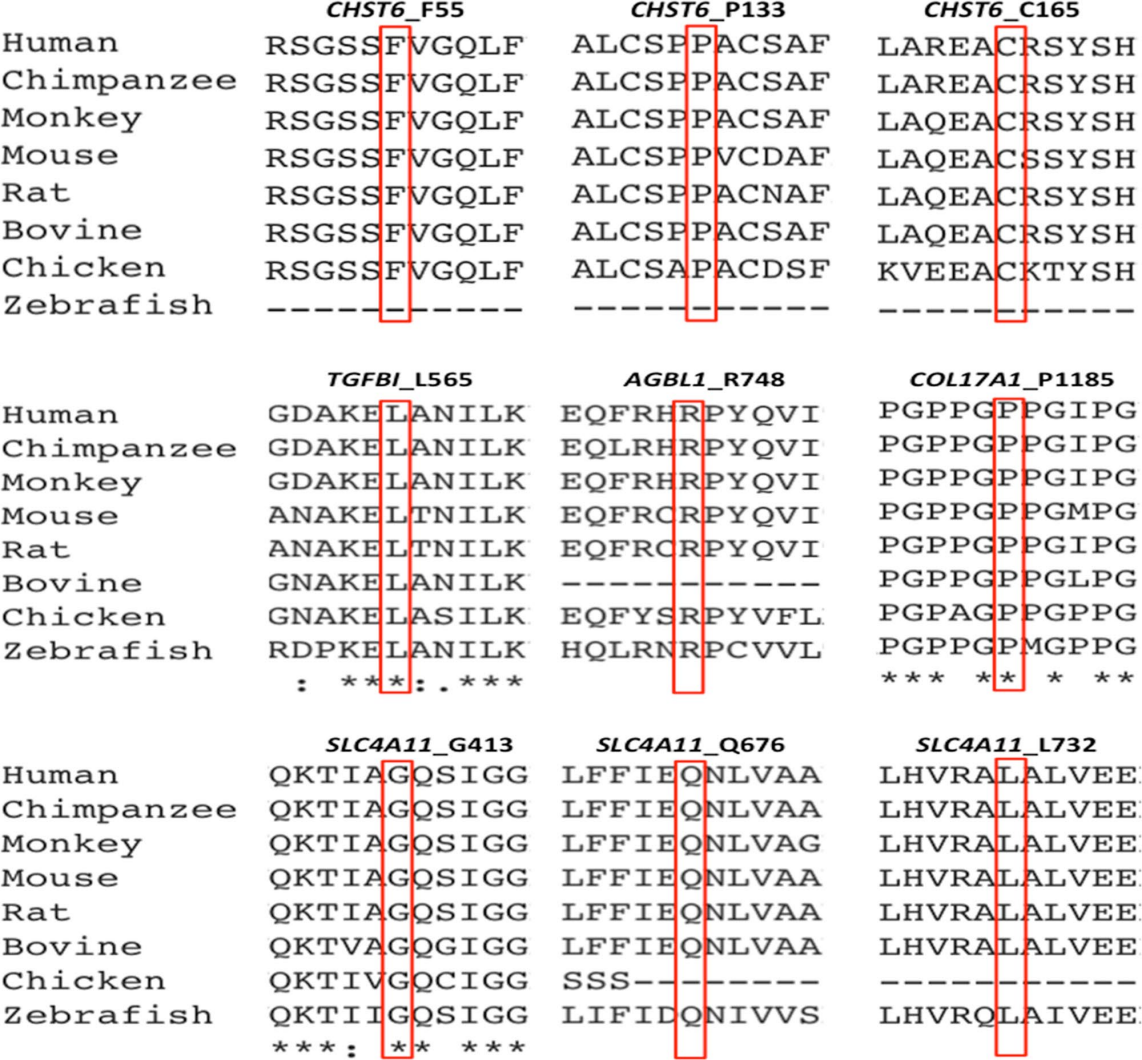
Conservation analysis of the nine novel mutations identified in this study.

Most cases of MCD caused by a CHST6 deficiency exhibited a recessive pattern of inheritance, although the phenotype caused by a single heterozygous mutation in CHST6 has also been reported previously (Aldave et al., 2004; Nowinska et al., 2014b). In our recent publication that summarized all the reported CHST6 mutations from literature, we found that among the 408 reported MCD patients with pathogenic CHST6 variants, 40 of them carried only one single heterozygous mutation (Zhang et al., 2019). Consistently, in the present study, many of the MCD cases were related to homozygous or compound heterozygous mutations in CHST6 (60.9%, 14/23), while heterozygous mutations including four missense mutations at positions 55, 133, 205, and 211 as well as two nonsense mutations at positions 165 and 254 were also observed. It was quite possible that a second causal mutation was missed by previous sequencing methods; for example, it might be located in the noncoding regions, and thereby, whole genome sequencing should be applied to these cases. In addition, many of the heterozygous mutations are in the sulfate donor, that is, the 3’-phosphoadenosine-5’-phosphosulfate binding region (Akama et al., 2000), or in other regions that are highly conserved among sulfotransferase enzymes (El-Ashry et al., 2005). These mutations were predicted to be damaging by an in silico analysis (**Table 2**), and they were highly conserved among species (**Figure 5**). The p.P133R mutation is surrounded by several validated pathogenic mutations of MCD, such as p.S131L, p.S131P, p.P132L, and p.A134D, implying that it might be located in a mutation hotspot. In addition, missense mutations at position 165 were reported, including p.C165W and p.C165Y, while a truncated protein product caused by the novel nonsense mutation (p.C165X) may lead to a potentially severe abnormality of CHST6 function.

At least 60 mutations in TGFBI associated with CDs have been identified to date, and the disease affects multiple layers of the cornea (Han et al., 2016). Among these mutations, p.R124H is the most frequently reported mutation worldwide and is the most frequently observed genotype among Asian patients with CD (Lakshminarayanan et al., 2014). Consistent with these previous results, the largest number of cases in our study carried the p. R124H mutation. Interestingly, we identified a novel p.L565H mutation in two unrelated cases. They were not attributed to any specific CD type during the initial clinical diagnosis, since they both revealed slightly diffuse opacities and haze in the central cornea, and no typical shape of the deposits was observed. However, with the aid of their genetic screening results, we found that the clinical manifestations in these two patients were quite similar to a previous case report of atypical lattice CD caused by a p.L565P mutation at the same position. Our results provide additional evidence that TRS could provide significant diagnostic information in the setting of genetically heterogeneous CD.

Four FECD loci on chromosomes 13, 18, 5, and 9, along with some linkage peaks and susceptibility loci, have been identified to confer disease risk. In addition, mutations in a variety of genes have pathogenic roles in FECD, including ZEB1, COL8A2, SLC4A11, AGBL1, LOXHD1, TCF4, and FEN1 (Elhalis et al., 2010; Vedana et al., 2016). However, the genetic basis of FECD is complicated and still far from understood, demonstrating variable expressivity and incomplete penetrance. In the current study, most of the patients (13 out of 19) with undetermined genetic etiology suffered from FECD, implying that TRS is less effective for this specific type of CD. Thus, more work, such as whole-exome sequencing, is necessary to elucidate the genetic basis of FECD, including the exploration of linked susceptibility loci as well as novel genes involved in its pathogenesis.

Several limitations that are common for most TRS techniques should be noted. First, this study mainly focused on coding exons and UTRs of known candidate genes for eye diseases, while variation in intronic regions was not examined, except for the TCF4 gene. The detection of copy number variation, large insertions, and deletions is still problematic using this approach. Second, using this TRS approach, relevant genes not yet connected to CD or other ocular diseases may be missing from the analysis. In this study, 19 cases had no variants of interest, and further investigations are required to elucidate their genetic etiology, probably through whole-exome sequencing, whole-genome sequencing, or other approaches. Furthermore, the identification of likely pathogenic variants in patients with CD was mainly based on in silico analyses. Although the use of multiple computational prediction tools boosts the reliability of variant classification in many diseases (Pasupati et al., 2017; Zaki et al., 2017), the current study still lacks experimental evidence to verify their contributions to the pathogenesis of CDs. Therefore, further functional investigations focused on specific mutations are required to elucidate the underlying mechanisms.

## Conclusion

This study enriched the list of genetic mutations that are associated with various types of CDs in a Chinese population and highlights the diagnostic value of molecular genetic analysis for patients with CDs.

## Data Availability

The targeted DNA sequencing data can be found in FigShare database, with the following accession IDs: 10.6084/m9.figshare.9632867; 10.6084/m9.figshare.9636296.

## Ethics Statement

The whole research procedure was performed in accordance with the Declaration of Helsinki and was approved by the Ethics Committee of the Eye and ENT Hospital.

## Author Contributions

JX designed the study. JZ, DW, YL, and YF performed the experiments. JZ, DW, YL, HC, and JH analyzed the data. JZ wrote the original draft. JX, DW, YL, YF, HC, JH, and JX revised the manuscript.

## Funding

The authors were sponsored by the National Natural Science Foundation of China (81870630, 81700806, 81670820, 81670818) and the Natural Science Foundation of Shanghai (17ZR1404400). The sponsor or funding organization had no role in the design or conduct of this research.

## Conflict of Interest Statement

The authors declare that the research was conducted in the absence of any commercial or financial relationships that could be construed as a potential conflict of interest.

## Supplementary Material

Figure S1 Compound heterozygous mutations determined by parental genotype.
