# Supplementary figures and images for: Novel Mutations Associated With Various Types of Corneal Dystrophies in a Han Chinese Population
Source: Front Genet. 2019 Aug 29;10:881. doi: 10.3389/fgene.2019.00881 (PMC6726741; doi:10.3389/fgene.2019.00881)

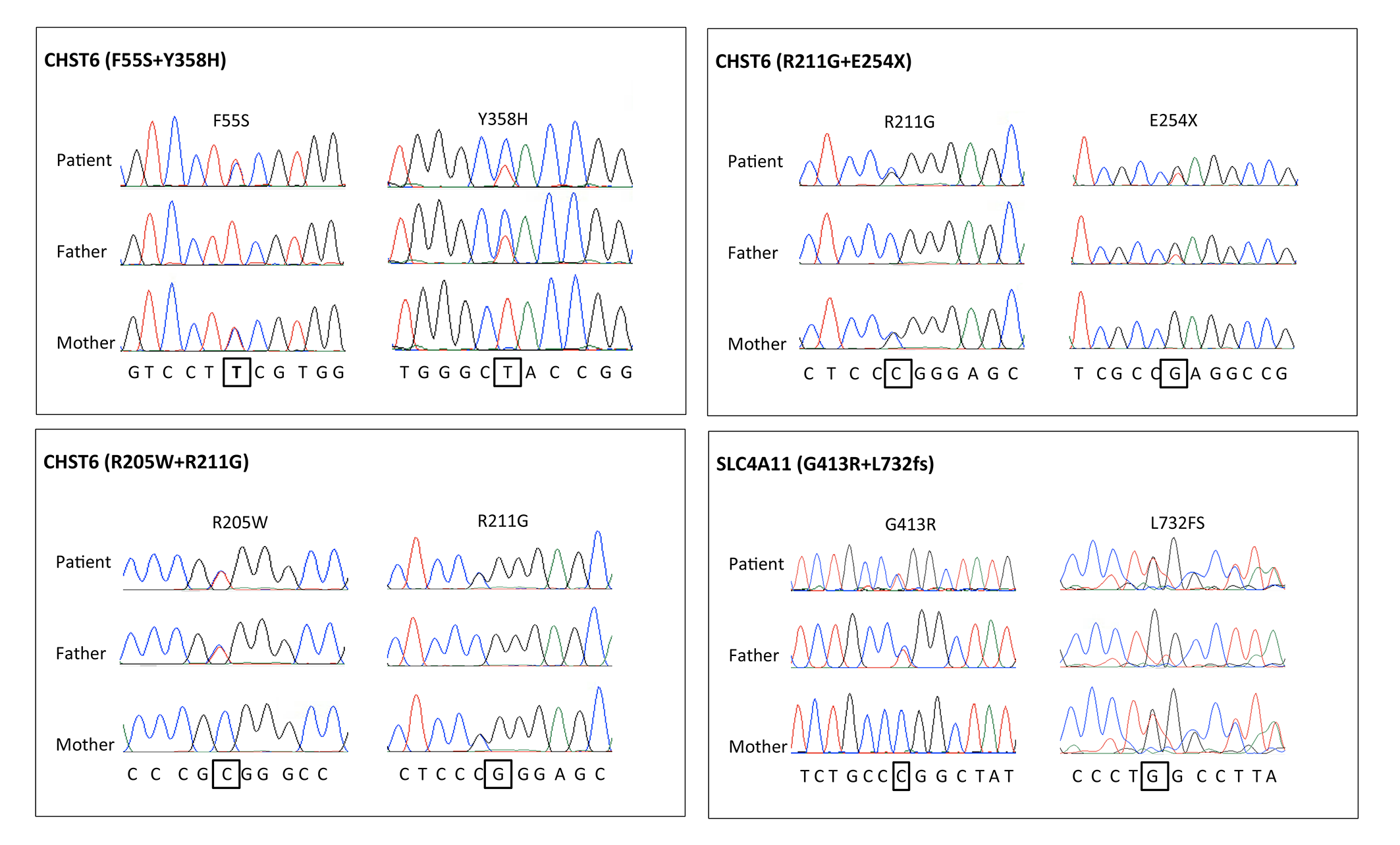

Supplement: Figure S1 — Compound heterozygous mutations determined by parental genotype. [file Image_1.tif]
