# Supplementary material for: Novel Mutations Associated With Various Types of Corneal Dystrophies in a Han Chinese Population
Source: Front Genet. 2019 Aug 29;10:881. doi: 10.3389/fgene.2019.00881 (PMC6726741; doi:10.3389/fgene.2019.00881)
Supplement: Supplementary file 2 [file DataSheet_1.pdf]

Table S1. 801 candidate genes of eye diseases included in TRS panel			
Ensembl Gene ID	EntrezGene ID	Associated Gene Name	Description
ENSG00000103375	343	AQP8	aquaporin 8 [Source:HGNC Symbol;Acc:HGNC:642]
ENSG00000160963	136227	COL26A1	collagen, type XXVI, alpha 1 [Source:HGNC Symbol;Acc:HGNC:18038]
ENSG00000275896	5645	PRSS2	protease, serine, 2 (trypsin 2) [Source:HGNC Symbol;Acc:HGNC:9483]
ENSG00000145476	285440	CYP4V2	cytochrome P450, family 4, subfamily V, polypeptide 2 [Source:HGNC Symbol;Acc:HGNC:23198]
ENSG00000121691	847	CAT	catalase [Source:HGNC Symbol;Acc:HGNC:1516]
ENSG00000138829	2201	FBN2	fibrillin 2 [Source:HGNC Symbol;Acc:HGNC:3604]
ENSG00000207695	406960	MIR184	microRNA 184 [Source:HGNC Symbol;Acc:HGNC:31555]
ENSG00000278695		GSTT2B	glutathione S-transferase theta 2B (gene/pseudogene) [Source:HGNC Symbol;Acc:HGNC:33437]
ENSG00000175040	9435	CHST2	carbohydrate (N-acetylglucosamine-6-O) sulfotransferase 2 [Source:HGNC Symbol;Acc:HGNC:1970]
ENSG00000101203	57642	COL20A1	collagen, type XX, alpha 1 [Source:HGNC Symbol;Acc:HGNC:14670]
ENSG00000161638	3678	ITGA5	integrin, alpha 5 (fibronectin receptor, alpha polypeptide) [Source:HGNC Symbol;Acc:HGNC:6141]
ENSG00000151694	6868	ADAM17	ADAM metallopeptidase domain 17 [Source:HGNC Symbol;Acc:HGNC:195]
ENSG00000113070	1839	HBEGF	heparin-binding EGF-like growth factor [Source:HGNC Symbol;Acc:HGNC:3059]
ENSG00000168542	1281	COL3A1	collagen, type III, alpha 1 [Source:HGNC Symbol;Acc:HGNC:2201]
ENSG00000120942	29914	UBIAD1	UbiA prenyltransferase domain containing 1 [Source:HGNC Symbol;Acc:HGNC:30791]
ENSG00000151914	667	DST	dystonin [Source:HGNC Symbol;Acc:HGNC:1090]
ENSG00000143387	1513	CTSK	cathepsin K [Source:HGNC Symbol;Acc:HGNC:2536]
ENSG00000135702	23563	CHST5	carbohydrate (N-acetylglucosamine 6-O) sulfotransferase 5 [Source:HGNC Symbol;Acc:HGNC:1973]
ENSG00000138685	2247	FGF2	fibroblast growth factor 2 (basic) [Source:HGNC Symbol;Acc:HGNC:3676]
ENSG00000100292	3162	HMOX1	heme oxygenase 1 [Source:HGNC Symbol;Acc:HGNC:5013]
ENSG00000164692	1278	COL1A2	collagen, type I, alpha 2 [Source:HGNC Symbol;Acc:HGNC:2198]

ENSG00000072210	224	ALDH3A2	aldehyde dehydrogenase 3 family, member A2 [Source:HGNC Symbol;Acc:HGNC:403]
ENSG00000124749	81578	COL21A1	collagen, type XXI, alpha 1 [Source:HGNC Symbol;Acc:HGNC:17025]
ENSG00000117525	2152	F3	coagulation factor III (thromboplastin, tissue factor) [Source:HGNC Symbol;Acc:HGNC:3541]
ENSG00000233276	2876	GPX1	glutathione peroxidase 1 [Source:HGNC Symbol;Acc:HGNC:4553]
ENSG00000100234	7078	TIMP3	TIMP metallopeptidase inhibitor 3 [Source:HGNC Symbol;Acc:HGNC:11822]
ENSG00000197930	30001	ERO1L	ERO1-like (S. cerevisiae) [Source:HGNC Symbol;Acc:HGNC:13280]
ENSG00000275199	10000	AKT3	v-akt murine thymoma viral oncogene homolog 3 [Source:HGNC Symbol;Acc:HGNC:393]
ENSG00000171819	10218	ANGPTL7	angiopoietin-like 7 [Source:HGNC Symbol;Acc:HGNC:24078]
ENSG00000102393	2717	GLA	galactosidase, alpha [Source:HGNC Symbol;Acc:HGNC:4296]
ENSG00000088926	2160	F11	coagulation factor XI [Source:HGNC Symbol;Acc:HGNC:3529]
ENSG00000148516	6935	ZEB1	zinc finger E-box binding homeobox 1 [Source:HGNC Symbol;Acc:HGNC:11642]
ENSG00000232541	1302	COL11A2	collagen, type XI, alpha 2 [Source:HGNC Symbol;Acc:HGNC:2187]
ENSG00000153936	9653	HS2ST1	heparan sulfate 2-O-sulfotransferase 1 [Source:HGNC Symbol;Acc:HGNC:5193]
ENSG00000154736	11096	ADAMTS5	ADAM metallopeptidase with thrombospondin type 1 motif, 5 [Source:HGNC Symbol;Acc:HGNC:221]
ENSG00000198001	51135	IRAK4	interleukin-1 receptor-associated kinase 4 [Source:HGNC Symbol;Acc:HGNC:17967]
ENSG00000125898	83541	FAM110A	family with sequence similarity 110, member A [Source:HGNC Symbol;Acc:HGNC:16188]
ENSG00000111799	1303	COL12A1	collagen, type XII, alpha 1 [Source:HGNC Symbol;Acc:HGNC:2188]
ENSG00000142208	207	AKT1	v-akt murine thymoma viral oncogene homolog 1 [Source:HGNC Symbol;Acc:HGNC:391]
ENSG00000120129	1843	DUSP1	dual specificity phosphatase 1 [Source:HGNC Symbol;Acc:HGNC:3064]
ENSG00000176153	2877	GPX2	glutathione peroxidase 2 [Source:HGNC Symbol;Acc:HGNC:4554]
ENSG00000166147	2200	FBN1	fibrillin 1 [Source:HGNC Symbol;Acc:HGNC:3603]
ENSG00000177426	7050	TGIF1	TGFB-induced factor homeobox 1 [Source:HGNC Symbol;Acc:HGNC:11776]
ENSG00000167210	125336	LOXHD1	lipoxygenase homology domains 1 [Source:HGNC Symbol;Acc:HGNC:26521]

ENSG00000165672	10935	PRDX3	peroxiredoxin 3 [Source:HGNC Symbol;Acc:HGNC:9354]
ENSG00000005156	3980	LIG3	ligase III, DNA, ATP-dependent [Source:HGNC Symbol;Acc:HGNC:6600]
ENSG00000185291	3563	IL3RA	interleukin 3 receptor, alpha (low affinity) [Source:HGNC Symbol;Acc:HGNC:6012]
ENSG00000145681	1404	HAPLN1	hyaluronan and proteoglycan link protein 1 [Source:HGNC Symbol;Acc:HGNC:2380]
ENSG00000145536	170690	ADAMTS16	ADAM metallopeptidase with thrombospondin type 1 motif, 16 [Source:HGNC Symbol;Acc:HGNC:17108]
ENSG00000171560	2243	FGA	fibrinogen alpha chain [Source:HGNC Symbol;Acc:HGNC:3661]
ENSG00000158859	9507	ADAMTS4	ADAM metallopeptidase with thrombospondin type 1 motif, 4 [Source:HGNC Symbol;Acc:HGNC:220]
ENSG00000126247	826	CAPNS1	calpain, small subunit 1 [Source:HGNC Symbol;Acc:HGNC:1481]
ENSG00000198734	2153	F5	coagulation factor V (proaccelerin, labile factor) [Source:HGNC Symbol;Acc:HGNC:3542]
ENSG00000003400	843	CASP10	caspase 10, apoptosis-related cysteine peptidase [Source:HGNC Symbol;Acc:HGNC:1500]
ENSG00000165806	840	CASP7	caspase 7, apoptosis-related cysteine peptidase [Source:HGNC Symbol;Acc:HGNC:1508]
ENSG00000137845	102	ADAM10	ADAM metallopeptidase domain 10 [Source:HGNC Symbol;Acc:HGNC:188]
ENSG00000182022	51363	CHST15	carbohydrate (N-acetylgalactosamine 4-sulfate 6-O) sulfotransferase 15 [Source:HGNC Symbol;Acc:HGNC:18137]
ENSG00000114251	7474	WNT5A	wingless-type MMTV integration site family, member 5A [Source:HGNC Symbol;Acc:HGNC:12784]
ENSG00000168036	1499	CTNNB1	catenin (cadherin-associated protein), beta 1, 88kDa [Source:HGNC Symbol;Acc:HGNC:2514]
ENSG00000282049	5645	PRSS2	protease, serine, 2 (trypsin 2) [Source:HGNC Symbol;Acc:HGNC:9483]
ENSG00000230930	1302	COL11A2	collagen, type XI, alpha 2 [Source:HGNC Symbol;Acc:HGNC:2187]
ENSG00000146648	1956	EGFR	epidermal growth factor receptor [Source:HGNC Symbol;Acc:HGNC:3236]
ENSG00000163132	4487	MSX1	msh homeobox 1 [Source:HGNC Symbol;Acc:HGNC:7391]
ENSG00000173599	5091	PC	pyruvate carboxylase [Source:HGNC Symbol;Acc:HGNC:8636]
ENSG00000115020	200576	PIKFYVE	phosphoinositide kinase, FYVE finger containing [Source:HGNC Symbol;Acc:HGNC:23785]

ENSG00000185010	2157	F8	coagulation factor VIII, procoagulant component [Source:HGNC Symbol;Acc:HGNC:3546]
ENSG00000051523	1535	CYBA	cytochrome b-245, alpha polypeptide [Source:HGNC Symbol;Acc:HGNC:2577]
ENSG00000084636	1307	COL16A1	collagen, type XVI, alpha 1 [Source:HGNC Symbol;Acc:HGNC:2193]
ENSG00000104365	3551	IKBKB	inhibitor of kappa light polypeptide gene enhancer in B-cells, kinase beta [Source:HGNC Symbol;Acc:HGNC:5960]
ENSG00000003402	8837	CFLAR	CASP8 and FADD-like apoptosis regulator [Source:HGNC Symbol;Acc:HGNC:1876]
ENSG00000087250	4504	MT3	metallothionein 3 [Source:HGNC Symbol;Acc:HGNC:7408]
ENSG00000277571	1991	ELANE	elastase, neutrophil expressed [Source:HGNC Symbol;Acc:HGNC:3309]
ENSG00000166748	123624	AGBL1	ATP/GTP binding protein-like 1 [Source:HGNC Symbol;Acc:HGNC:26504]
ENSG00000014216	823	CAPN1	calpain 1, (mu/I) large subunit [Source:HGNC Symbol;Acc:HGNC:1476]
ENSG00000165458	3636	INPPL1	inositol polyphosphate phosphatase-like 1 [Source:HGNC Symbol;Acc:HGNC:6080]
ENSG00000204291	1306	COL15A1	collagen, type XV, alpha 1 [Source:HGNC Symbol;Acc:HGNC:2192]
ENSG00000184292	4070	TACSTD2	tumor-associated calcium signal transducer 2 [Source:HGNC Symbol;Acc:HGNC:11530]
ENSG00000171552	598	BCL2L1	BCL2-like 1 [Source:HGNC Symbol;Acc:HGNC:992]
ENSG00000066032	1496	CTNNA2	catenin (cadherin-associated protein), alpha 2 [Source:HGNC Symbol;Acc:HGNC:2510]
ENSG00000049089	1298	COL9A2	collagen, type IX, alpha 2 [Source:HGNC Symbol;Acc:HGNC:2218]
ENSG00000118113	4317	MMP8	matrix metallopeptidase 8 [Source:HGNC Symbol;Acc:HGNC:7175]
ENSG00000172115	54205	CYCS	cytochrome c, somatic [Source:HGNC Symbol;Acc:HGNC:19986]
ENSG00000226071	3116	HLA-DPB2	major histocompatibility complex, class II, DP beta 2 (pseudogene) [Source:HGNC Symbol;Acc:HGNC:4941]
ENSG00000171791	596	BCL2	B-cell CLL/lymphoma 2 [Source:HGNC Symbol;Acc:HGNC:990]
ENSG00000171502	255631	COL24A1	collagen, type XXIV, alpha 1 [Source:HGNC Symbol;Acc:HGNC:20821]
ENSG00000148848	8038	ADAM12	ADAM metallopeptidase domain 12 [Source:HGNC Symbol;Acc:HGNC:190]
ENSG00000164294	493869	GPX8	glutathione peroxidase 8 (putative) [Source:HGNC Symbol;Acc:HGNC:33100]

ENSG00000083782	1833	EPYC	epiphycan [Source:HGNC Symbol;Acc:HGNC:3053]
ENSG00000164823	734	OSGIN2	oxidative stress induced growth inhibitor family member 2 [Source:HGNC Symbol;Acc:HGNC:1355]
ENSG00000149311	472	ATM	ATM serine/threonine kinase [Source:HGNC Symbol;Acc:HGNC:795]
ENSG00000125398	6662	SOX9	SRY (sex determining region Y)-box 9 [Source:HGNC Symbol;Acc:HGNC:11204]
ENSG00000120708	7045	TGFBI	transforming growth factor, beta-induced, 68kDa [Source:HGNC Symbol;Acc:HGNC:11771]
ENSG00000140632	84656	GLYR1	glyoxylate reductase 1 homolog (Arabidopsis) [Source:HGNC Symbol;Acc:HGNC:24434]
ENSG00000182871	80781	COL18A1	collagen, type XVIII, alpha 1 [Source:HGNC Symbol;Acc:HGNC:2195]
ENSG00000132518	3000	GUCY2D	guanylate cyclase 2D, membrane (retina-specific) [Source:HGNC Symbol;Acc:HGNC:4689]
ENSG00000087088	581	BAX	BCL2-associated X protein [Source:HGNC Symbol;Acc:HGNC:959]
ENSG00000091409	3655	ITGA6	integrin, alpha 6 [Source:HGNC Symbol;Acc:HGNC:6142]
ENSG00000140443	3480	IGF1R	insulin-like growth factor 1 receptor [Source:HGNC Symbol;Acc:HGNC:5465]
ENSG00000141556	6904	TBCD	tubulin folding cofactor D [Source:HGNC Symbol;Acc:HGNC:11581]
ENSG00000166670	4319	MMP10	matrix metallopeptidase 10 [Source:HGNC Symbol;Acc:HGNC:7156]
ENSG00000198756	23127	COLGALT2	collagen beta(1-O)galactosyltransferase 2 [Source:HGNC Symbol;Acc:HGNC:16790]
ENSG00000105664	1311	COMP	cartilage oligomeric matrix protein [Source:HGNC Symbol;Acc:HGNC:2227]
ENSG00000163331	92196	DAPL1	death associated protein-like 1 [Source:HGNC Symbol;Acc:HGNC:21490]
ENSG00000115414	2335	FN1	fibronectin 1 [Source:HGNC Symbol;Acc:HGNC:3778]
ENSG00000135424	3679	ITGA7	integrin, alpha 7 [Source:HGNC Symbol;Acc:HGNC:6143]
ENSG00000060718	1301	COL11A1	collagen, type XI, alpha 1 [Source:HGNC Symbol;Acc:HGNC:2186]
ENSG00000196954	837	CASP4	caspase 4, apoptosis-related cysteine peptidase [Source:HGNC Symbol;Acc:HGNC:1505]
ENSG00000250510	27239	GPR162	G protein-coupled receptor 162 [Source:HGNC Symbol;Acc:HGNC:16693]
ENSG00000117984	1509	CTSD	cathepsin D [Source:HGNC Symbol;Acc:HGNC:2529]
ENSG00000137273	2295	FOXF2	forkhead box F2 [Source:HGNC Symbol;Acc:HGNC:3810]

ENSG00000168925	1504	CTRB1	chymotrypsinogen B1 [Source:HGNC Symbol;Acc:HGNC:2521]
ENSG00000173992	9973	CCS	copper chaperone for superoxide dismutase [Source:HGNC Symbol;Acc:HGNC:1613]
ENSG00000187955	7373	COL14A1	collagen, type XIV, alpha 1 [Source:HGNC Symbol;Acc:HGNC:2191]
ENSG00000282230	8754	ADAM9	ADAM metallopeptidase domain 9 [Source:HGNC Symbol;Acc:HGNC:216]
ENSG00000138448	3685	ITGAV	integrin, alpha V [Source:HGNC Symbol;Acc:HGNC:6150]
ENSG00000132906	842	CASP9	caspase 9, apoptosis-related cysteine peptidase [Source:HGNC Symbol;Acc:HGNC:1511]
ENSG00000169031	1285	COL4A3	collagen, type IV, alpha 3 (Goodpasture antigen) [Source:HGNC Symbol;Acc:HGNC:2204]
ENSG00000198650	6898	TAT	tyrosine aminotransferase [Source:HGNC Symbol;Acc:HGNC:11573]
ENSG00000081052	1286	COL4A4	collagen, type IV, alpha 4 [Source:HGNC Symbol;Acc:HGNC:2206]
ENSG00000263243	3859	KRT12	keratin 12, type I [Source:HGNC Symbol;Acc:HGNC:6414]
ENSG00000084207	2950	GSTP1	glutathione S-transferase pi 1 [Source:HGNC Symbol;Acc:HGNC:4638]
ENSG00000143278	2165	F13B	coagulation factor XIII, B polypeptide [Source:HGNC Symbol;Acc:HGNC:3534]
ENSG00000248746	89	ACTN3	actinin, alpha 3 (gene/pseudogene) [Source:HGNC Symbol;Acc:HGNC:165]
ENSG00000156140	9508	ADAMTS3	ADAM metallopeptidase with thrombospondin type 1 motif, 3 [Source:HGNC Symbol;Acc:HGNC:219]
ENSG00000139330	11081	KERA	keratocan [Source:HGNC Symbol;Acc:HGNC:6309]
ENSG00000213949	3672	ITGA1	integrin, alpha 1 [Source:HGNC Symbol;Acc:HGNC:6134]
ENSG00000187498	1282	COL4A1	collagen, type IV, alpha 1 [Source:HGNC Symbol;Acc:HGNC:2202]
ENSG00000100985	4318	MMP9	matrix metallopeptidase 9 [Source:HGNC Symbol;Acc:HGNC:7176]
ENSG00000135047	1514	CTSL	cathepsin L [Source:HGNC Symbol;Acc:HGNC:2537]
ENSG00000167244	3481	IGF2	insulin-like growth factor 2 [Source:HGNC Symbol;Acc:HGNC:5466]
ENSG00000163359	1293	COL6A3	collagen, type VI, alpha 3 [Source:HGNC Symbol;Acc:HGNC:2213]
ENSG00000164733	1508	CTSB	cathepsin B [Source:HGNC Symbol;Acc:HGNC:2527]
ENSG00000278759	6904	TBCD	tubulin folding cofactor D [Source:HGNC Symbol;Acc:HGNC:11581]
ENSG00000171812	1296	COL8A2	collagen, type VIII, alpha 2 [Source:HGNC Symbol;Acc:HGNC:2216]

ENSG00000137332	780	DDR1	discoidin domain receptor tyrosine kinase 1 [Source:HGNC Symbol;Acc:HGNC:2730]
ENSG00000187242	3859	KRT12	keratin 12, type I [Source:HGNC Symbol;Acc:HGNC:6414]
ENSG00000163098	112401	BIRC8	baculoviral IAP repeat containing 8 [Source:HGNC Symbol;Acc:HGNC:14878]
ENSG00000211445	2878	GPX3	glutathione peroxidase 3 [Source:HGNC Symbol;Acc:HGNC:4555]
ENSG00000206384	131873	COL6A6	collagen, type VI, alpha 6 [Source:HGNC Symbol;Acc:HGNC:27023]
ENSG00000116701	4688	NCF2	neutrophil cytosolic factor 2 [Source:HGNC Symbol;Acc:HGNC:7661]
ENSG00000026508	960	CD44	CD44 molecule (Indian blood group) [Source:HGNC Symbol;Acc:HGNC:1681]
ENSG00000136943	1515	CTSV	cathepsin V [Source:HGNC Symbol;Acc:HGNC:2538]
ENSG00000221823	5534	PPP3R1	protein phosphatase 3, regulatory subunit B, alpha [Source:HGNC Symbol;Acc:HGNC:9317]
ENSG00000164399	3562	IL3	interleukin 3 [Source:HGNC Symbol;Acc:HGNC:6011]
ENSG00000163131	1520	CTSS	cathepsin S [Source:HGNC Symbol;Acc:HGNC:2545]
ENSG00000123500	1300	COL10A1	collagen, type X, alpha 1 [Source:HGNC Symbol;Acc:HGNC:2185]
ENSG00000115008	3552	IL1A	interleukin 1, alpha [Source:HGNC Symbol;Acc:HGNC:5991]
ENSG00000090376	11213	IRAK3	interleukin-1 receptor-associated kinase 3 [Source:HGNC Symbol;Acc:HGNC:17020]
ENSG00000100823	328	APEX1	APEX nuclease (multifunctional DNA repair enzyme) 1 [Source:HGNC Symbol;Acc:HGNC:587]
ENSG00000140873	170692	ADAMTS18	ADAM metallopeptidase with thrombospondin type 1 motif, 18 [Source:HGNC Symbol;Acc:HGNC:17110]
ENSG00000018236	1272	CNTN1	contactin 1 [Source:HGNC Symbol;Acc:HGNC:2171]
ENSG00000164171	3673	ITGA2	integrin, alpha 2 (CD49B, alpha 2 subunit of VLA-2 receptor) [Source:HGNC Symbol;Acc:HGNC:6137]
ENSG00000168379	3116	HLA-DPB2	major histocompatibility complex, class II, DP beta 2 (pseudogene) [Source:HGNC Symbol;Acc:HGNC:4941]
ENSG00000134871	1284	COL4A2	collagen, type IV, alpha 2 [Source:HGNC Symbol;Acc:HGNC:2203]
ENSG00000144724	5793	PTPRG	protein tyrosine phosphatase, receptor type, G [Source:HGNC Symbol;Acc:HGNC:9671]
ENSG00000168615	8754	ADAM9	ADAM metallopeptidase domain 9 [Source:HGNC Symbol;Acc:HGNC:216]

ENSG00000017427	3479	IGF1	insulin-like growth factor 1 (somatomedin C) [Source:HGNC Symbol;Acc:HGNC:5464]
ENSG00000125931	4435	CITED1	Cbp/p300-interacting transactivator, with Glu/Asp-rich carboxy-terminal domain, 1 [Source:HGNC Symbol;Acc:HGNC:1986]
ENSG00000175899	2	A2M	alpha-2-macroglobulin [Source:HGNC Symbol;Acc:HGNC:7]
ENSG00000100368	1439	CSF2RB	colony stimulating factor 2 receptor, beta, low-affinity (granulocyte-macrophage) [Source:HGNC Symbol;Acc:HGNC:2436]
ENSG00000114270	1294	COL7A1	collagen, type VII, alpha 1 [Source:HGNC Symbol;Acc:HGNC:2214]
ENSG00000223680	780	DDR1	discoidin domain receptor tyrosine kinase 1 [Source:HGNC Symbol;Acc:HGNC:2730]
ENSG00000134376	23418	CRB1	crumbs family member 1, photoreceptor morphogenesis associated [Source:HGNC Symbol;Acc:HGNC:2343]
ENSG00000170558	1000	CDH2	cadherin 2, type 1, N-cadherin (neuronal) [Source:HGNC Symbol;Acc:HGNC:1759]
ENSG00000115474	3769	KCNJ13	potassium channel, inwardly rectifying subfamily J, member 13 [Source:HGNC Symbol;Acc:HGNC:6259]
ENSG00000197451	3182	HNRNPAB	heterogeneous nuclear ribonucleoprotein A/B [Source:HGNC Symbol;Acc:HGNC:5034]
ENSG00000177697	977	CD151	CD151 molecule (Raph blood group) [Source:HGNC Symbol;Acc:HGNC:1630]
ENSG00000137757	838	CASP5	caspase 5, apoptosis-related cysteine peptidase [Source:HGNC
ENSG00000138795	51176	LEF1	lymphoid enhancer-binding factor 1 [Source:HGNC Symbol;Acc:HGNC:6551]
ENSG00000064012	841	CASP8	caspase 8, apoptosis-related cysteine peptidase [Source:HGNC Symbol;Acc:HGNC:1509]
ENSG00000139329	4060	LUM	lumican [Source:HGNC Symbol;Acc:HGNC:6724]
ENSG00000167136	2021	ENDOG	endonuclease G [Source:HGNC Symbol;Acc:HGNC:3346]
ENSG00000155760	8324	FZD7	frizzled class receptor 7 [Source:HGNC Symbol;Acc:HGNC:4045]
ENSG00000149564	90952	ESAM	endothelial cell adhesion molecule [Source:HGNC Symbol;Acc:HGNC:17474]
ENSG00000142798	3339	HSPG2	heparan sulfate proteoglycan 2 [Source:HGNC Symbol;Acc:HGNC:5273]
ENSG00000100987	30813	VSX1	visual system homeobox 1 [Source:HGNC Symbol;Acc:HGNC:12723]
ENSG00000196739	85301	COL27A1	collagen, type XXVII, alpha 1 [Source:HGNC Symbol;Acc:HGNC:22986]

ENSG00000129221	23746	AIPL1	aryl hydrocarbon receptor interacting protein-like 1 [Source:HGNC Symbol;Acc:HGNC:359]
ENSG00000057593	2155	F7	coagulation factor VII (serum prothrombin conversion accelerator) [Source:HGNC Symbol;Acc:HGNC:3544]
ENSG00000015475	637	BID	BH3 interacting domain death agonist [Source:HGNC Symbol;Acc:HGNC:1050]
ENSG00000175264	8534	CHST1	carbohydrate (keratan sulfate Gal-6) sulfotransferase 1 [Source:HGNC Symbol;Acc:HGNC:1969]
ENSG00000170275	10491	CRTAP	cartilage associated protein [Source:HGNC Symbol;Acc:HGNC:2379]
ENSG00000197565	1288	COL4A6	collagen, type IV, alpha 6 [Source:HGNC Symbol;Acc:HGNC:2208]
ENSG00000142449	84467	FBN3	fibrillin 3 [Source:HGNC Symbol;Acc:HGNC:18794]
ENSG00000117560	356	FASLG	Fas ligand (TNF superfamily, member 6) [Source:HGNC Symbol;Acc:HGNC:11936]
ENSG00000172270	682	BSG	basigin (Ok blood group) [Source:HGNC Symbol;Acc:HGNC:1116]
ENSG00000139219	1280	COL2A1	collagen, type II, alpha 1 [Source:HGNC Symbol;Acc:HGNC:2200]
ENSG00000178568	2066	ERBB4	erb-b2 receptor tyrosine kinase 4 [Source:HGNC Symbol;Acc:HGNC:3432]
ENSG00000039068	999	CDH1	cadherin 1, type 1, E-cadherin (epithelial) [Source:HGNC Symbol;Acc:HGNC:1748]
ENSG00000234078	780	DDR1	discoidin domain receptor tyrosine kinase 1 [Source:HGNC Symbol;Acc:HGNC:2730]
ENSG00000240583	358	AQP1	aquaporin 1 (Colton blood group) [Source:HGNC Symbol;Acc:HGNC:633]
ENSG00000196154	6275	S100A4	S100 calcium binding protein A4 [Source:HGNC Symbol;Acc:HGNC:10494]
ENSG00000126432	25824	PRDX5	peroxiredoxin 5 [Source:HGNC Symbol;Acc:HGNC:9355]
ENSG00000120868	317	APAF1	apoptotic peptidase activating factor 1 [Source:HGNC Symbol;Acc:HGNC:576]
ENSG00000171557	2266	FGG	fibrinogen gamma chain [Source:HGNC Symbol;Acc:HGNC:3694]
ENSG00000143537	8751	ADAM15	ADAM metallopeptidase domain 15 [Source:HGNC Symbol;Acc:HGNC:193]
ENSG00000147044	8573	CASK	calcium/calmodulin-dependent serine protein kinase (MAGUK family) [Source:HGNC Symbol;Acc:HGNC:1497]
ENSG00000198947	1756	DMD	dystrophin [Source:HGNC Symbol;Acc:HGNC:2928]
ENSG00000233584	3116	HLA-DPB2	major histocompatibility complex, class II, DP beta 2 (pseudogene) [Source:HGNC Symbol;Acc:HGNC:4941]

ENSG00000229767	780	DDR1	discoidin domain receptor tyrosine kinase 1 [Source:HGNC Symbol;Acc:HGNC:2730]
ENSG00000213341	1147	CHUK	conserved helix-loop-helix ubiquitous kinase [Source:HGNC Symbol;Acc:HGNC:1974]
ENSG00000188157	375790	AGRN	agrin [Source:HGNC Symbol;Acc:HGNC:329]
ENSG00000196083	3556	IL1RAP	interleukin 1 receptor accessory protein [Source:HGNC Symbol;Acc:HGNC:5995]
ENSG00000197467	1305	COL13A1	collagen, type XIII, alpha 1 [Source:HGNC Symbol;Acc:HGNC:2190]
ENSG00000106819	54829	ASPN	asporin [Source:HGNC Symbol;Acc:HGNC:14872]
ENSG00000002330	572	BAD	BCL2-associated agonist of cell death [Source:HGNC Symbol;Acc:HGNC:936]
ENSG00000162909	824	CAPN2	calpain 2, (m/II) large subunit [Source:HGNC Symbol;Acc:HGNC:1479]
ENSG00000215018	340267	COL28A1	collagen, type XXVIII, alpha 1 [Source:HGNC Symbol;Acc:HGNC:22442]
ENSG00000168487	649	BMP1	bone morphogenetic protein 1 [Source:HGNC Symbol;Acc:HGNC:1067]
ENSG00000108821	1277	COL1A1	collagen, type I, alpha 1 [Source:HGNC Symbol;Acc:HGNC:2197]
ENSG00000223372	3116	HLA-DPB2	major histocompatibility complex, class II, DP beta 2 (pseudogene) [Source:HGNC Symbol;Acc:HGNC:4941]
ENSG00000092969	7042	TGFB2	transforming growth factor, beta 2 [Source:HGNC Symbol;Acc:HGNC:11768]
ENSG00000125845	650	BMP2	bone morphogenetic protein 2 [Source:HGNC Symbol;Acc:HGNC:1069]
ENSG00000050767	91522	COL23A1	collagen, type XXIII, alpha 1 [Source:HGNC Symbol;Acc:HGNC:22990]
ENSG00000103569	366	AQP9	aquaporin 9 [Source:HGNC Symbol;Acc:HGNC:643]
ENSG00000180210	2147	F2	coagulation factor II (thrombin) [Source:HGNC Symbol;Acc:HGNC:3535]
ENSG00000225132	3116	HLA-DPB2	major histocompatibility complex, class II, DP beta 2 (pseudogene) [Source:HGNC Symbol;Acc:HGNC:4941]
ENSG00000116157	2882	GPX7	glutathione peroxidase 7 [Source:HGNC Symbol;Acc:HGNC:4559]
ENSG00000162594	149233	IL23R	interleukin 23 receptor [Source:HGNC Symbol;Acc:HGNC:19100]
ENSG00000051180	5888	RAD51	RAD51 recombinase [Source:HGNC Symbol;Acc:HGNC:9817]
ENSG00000136720	9394	HS6ST1	heparan sulfate 6-O-sulfotransferase 1 [Source:HGNC Symbol;Acc:HGNC:5201]
ENSG00000122176	2331	FMOD	fibromodulin [Source:HGNC Symbol;Acc:HGNC:3774]

ENSG00000131187	2161	F12	coagulation factor XII (Hageman factor) [Source:HGNC Symbol;Acc:HGNC:3530]
ENSG00000072110	87	ACTN1	actinin, alpha 1 [Source:HGNC Symbol;Acc:HGNC:163]
ENSG00000234834	3116	HLA-DPB2	major histocompatibility complex, class II, DP beta 2 (pseudogene) [Source:HGNC Symbol;Acc:HGNC:4941]
ENSG00000168928	440387	CTRB2	chymotrypsinogen B2 [Source:HGNC Symbol;Acc:HGNC:2522]
ENSG00000188517	84570	COL25A1	collagen, type XXV, alpha 1 [Source:HGNC Symbol;Acc:HGNC:18603]
ENSG00000092009	1215	CMA1	chymase 1, mast cell [Source:HGNC Symbol;Acc:HGNC:2097]
ENSG00000101197	79444	BIRC7	baculoviral IAP repeat containing 7 [Source:HGNC Symbol;Acc:HGNC:13702]
ENSG00000204403	1E+08	CASP12	caspase 12 (gene/pseudogene) [Source:HGNC Symbol;Acc:HGNC:19004]
ENSG00000080573	50509	COL5A3	collagen, type V, alpha 3 [Source:HGNC Symbol;Acc:HGNC:14864]
ENSG00000100644	3091	HIF1A	hypoxia inducible factor 1, alpha subunit (basic helix-loop-helix transcription factor) [Source:HGNC Symbol;Acc:HGNC:4910]
ENSG00000204248	1302	COL11A2	collagen, type XI, alpha 2 [Source:HGNC Symbol;Acc:HGNC:2187]
ENSG00000132692	63827	BCAN	brevican [Source:HGNC Symbol;Acc:HGNC:23059]
ENSG00000004961	3052	HCCS	holocytochrome c synthase [Source:HGNC Symbol;Acc:HGNC:4837]
ENSG00000224557	3116	HLA-DPB2	major histocompatibility complex, class II, DP beta 2 (pseudogene) [Source:HGNC Symbol;Acc:HGNC:4941]
ENSG00000140678	3687	ITGAX	integrin, alpha X (complement component 3 receptor 4 subunit) [Source:HGNC Symbol;Acc:HGNC:6152]
ENSG00000101846	412	STS	steroid sulfatase (microsomal), isozyme S [Source:HGNC Symbol;Acc:HGNC:11425]
ENSG00000082293	1310	COL19A1	collagen, type XIX, alpha 1 [Source:HGNC Symbol;Acc:HGNC:2196]
ENSG00000026103	355	FAS	Fas cell surface death receptor [Source:HGNC Symbol;Acc:HGNC:11920]
ENSG00000168040	8772	FADD	Fas (TNFRSF6)-associated via death domain [Source:HGNC Symbol;Acc:HGNC:3573]
ENSG00000124762	1026	CDKN1A	cyclin-dependent kinase inhibitor 1A (p21, Cip1) [Source:HGNC Symbol;Acc:HGNC:1784]
ENSG00000171551	9427	ECEL1	endothelin converting enzyme-like 1 [Source:HGNC Symbol;Acc:HGNC:3147]

ENSG00000171004	90161	HS6ST2	heparan sulfate 6-O-sulfotransferase 2 [Source:HGNC Symbol;Acc:HGNC:19133]
ENSG00000196628	6925	TCF4	transcription factor 4 [Source:HGNC Symbol;Acc:HGNC:11634]
ENSG00000183196	4166	CHST6	carbohydrate (N-acetylglucosamine 6-O) sulfotransferase 6 [Source:HGNC Symbol;Acc:HGNC:6938]
ENSG00000088836	83959	SLC4A11	solute carrier family 4, sodium borate transporter, member 11 [Source:HGNC Symbol;Acc:HGNC:16438]
ENSG00000140465	1543	CYP1A1	cytochrome P450, family 1, subfamily A, polypeptide 1 [Source:HGNC Symbol;Acc:HGNC:2595]
ENSG00000188375	440093	H3F3C	H3 histone, family 3C [Source:HGNC Symbol;Acc:HGNC:33164]
ENSG00000150093	3688	ITGB1	integrin, beta 1 (fibronectin receptor, beta polypeptide, antigen CD29 includes MDF2, MSK12) [Source:HGNC Symbol;Acc:HGNC:6153]
ENSG00000140522	6017	RLBP1	retinaldehyde binding protein 1 [Source:HGNC Symbol;Acc:HGNC:10024]
ENSG00000007372	5080	PAX6	paired box 6 [Source:HGNC Symbol;Acc:HGNC:8620]
ENSG00000188153	1287	COL4A5	collagen, type IV, alpha 5 [Source:HGNC Symbol;Acc:HGNC:2207]
ENSG00000164111	308	ANXA5	annexin A5 [Source:HGNC Symbol;Acc:HGNC:543]
ENSG00000126218	2159	F10	coagulation factor X [Source:HGNC Symbol;Acc:HGNC:3528]
ENSG00000019991	3082	HGF	hepatocyte growth factor (hepapoietin A; scatter factor) [Source:HGNC Symbol;Acc:HGNC:4893]
ENSG00000092758	1299	COL9A3	collagen, type IX, alpha 3 [Source:HGNC Symbol;Acc:HGNC:2219]
ENSG00000154864	63895	PIEZO2	piezo-type mechanosensitive ion channel component 2 [Source:HGNC Symbol;Acc:HGNC:26270]
ENSG00000118137	335	APOA1	apolipoprotein A-I [Source:HGNC Symbol;Acc:HGNC:600]
ENSG00000164305	836	CASP3	caspase 3, apoptosis-related cysteine peptidase [Source:HGNC Symbol;Acc:HGNC:1504]
ENSG00000176171	664	BNIP3	BCL2/adenovirus E1B 19kDa interacting protein 3 [Source:HGNC Symbol;Acc:HGNC:1084]
ENSG00000162733	4921	DDR2	discoidin domain receptor tyrosine kinase 2 [Source:HGNC Symbol;Acc:HGNC:2731]
ENSG00000183230	29119	CTNNA3	catenin (cadherin-associated protein), alpha 3 [Source:HGNC Symbol;Acc:HGNC:2511]

ENSG00000082701	2932	GSK3B	glycogen synthase kinase 3 beta [Source:HGNC Symbol;Acc:HGNC:4617]
ENSG00000125538	3553	IL1B	interleukin 1, beta [Source:HGNC Symbol;Acc:HGNC:5992]
ENSG00000167815	7001	PRDX2	peroxiredoxin 2 [Source:HGNC Symbol;Acc:HGNC:9353]
ENSG00000262406	4321	MMP12	matrix metallopeptidase 12 [Source:HGNC Symbol;Acc:HGNC:7158]
ENSG00000215522	780	DDR1	discoidin domain receptor tyrosine kinase 1 [Source:HGNC Symbol;Acc:HGNC:2730]
ENSG00000040531	1497	CTNS	cystinosin, lysosomal cystine transporter [Source:HGNC Symbol;Acc:HGNC:2518]
ENSG00000134917	11095	ADAMTS8	ADAM metallopeptidase with thrombospondin type 1 motif, 8 [Source:HGNC Symbol;Acc:HGNC:224]
ENSG00000112280	1297	COL9A1	collagen, type IX, alpha 1 [Source:HGNC Symbol;Acc:HGNC:2217]
ENSG00000227801	1302	COL11A2	collagen, type XI, alpha 2 [Source:HGNC Symbol;Acc:HGNC:2187]
ENSG00000065618	1308	COL17A1	collagen, type XVII, alpha 1 [Source:HGNC Symbol;Acc:HGNC:2194]
ENSG00000170345	2353	FOS	FBJ murine osteosarcoma viral oncogene homolog [Source:HGNC Symbol;Acc:HGNC:3796]
ENSG00000091513	7018	TF	transferrin [Source:HGNC Symbol;Acc:HGNC:11740]
ENSG00000101144	655	BMP7	bone morphogenetic protein 7 [Source:HGNC Symbol;Acc:HGNC:1074]
ENSG00000130726	10155	TRIM28	tripartite motif containing 28 [Source:HGNC Symbol;Acc:HGNC:16384]
ENSG00000157766	176	ACAN	aggrecan [Source:HGNC Symbol;Acc:HGNC:319]
ENSG00000172752	256076	COL6A5	collagen, type VI, alpha 5 [Source:HGNC Symbol;Acc:HGNC:26674]
ENSG00000124216	6615	SNAI1	snail family zinc finger 1 [Source:HGNC Symbol;Acc:HGNC:11128]
ENSG00000140564	5045	FURIN	furin (paired basic amino acid cleaving enzyme) [Source:HGNC Symbol;Acc:HGNC:8568]
ENSG00000105221	208	AKT2	v-akt murine thymoma viral oncogene homolog 2 [Source:HGNC Symbol;Acc:HGNC:392]
ENSG00000144668	3680	ITGA9	integrin, alpha 9 [Source:HGNC Symbol;Acc:HGNC:6145]
ENSG00000168496	2237	FEN1	flap structure-specific endonuclease 1 [Source:HGNC Symbol;Acc:HGNC:3650]
ENSG00000197561	1991	ELANE	elastase, neutrophil expressed [Source:HGNC Symbol;Acc:HGNC:3309]
ENSG00000117592	9588	PRDX6	peroxiredoxin 6 [Source:HGNC Symbol;Acc:HGNC:16753]

ENSG00000130309	79709	COLGALT1	collagen beta(1-O)galactosyltransferase 1 [Source:HGNC Symbol;Acc:HGNC:26182]
ENSG00000118689	2309	FOXO3	forkhead box O3 [Source:HGNC Symbol;Acc:HGNC:3821]
ENSG00000120885	1191	CLU	clusterin [Source:HGNC Symbol;Acc:HGNC:2095]
ENSG00000023445	330	BIRC3	baculoviral IAP repeat containing 3 [Source:HGNC Symbol;Acc:HGNC:591]
ENSG00000160049	1676	DFFA	DNA fragmentation factor, 45kDa, alpha polypeptide [Source:HGNC Symbol;Acc:HGNC:2772]
ENSG00000235708	1302	COL11A2	collagen, type XI, alpha 2 [Source:HGNC Symbol;Acc:HGNC:2187]
ENSG00000092820	7430	EZR	ezrin [Source:HGNC Symbol;Acc:HGNC:12691]
ENSG00000152592	1758	DMP1	dentin matrix acidic phosphoprotein 1 [Source:HGNC Symbol;Acc:HGNC:2932]
ENSG00000152591	1834	DSPP	dentin sialophosphoprotein [Source:HGNC Symbol;Acc:HGNC:3054]
ENSG00000110330	329	BIRC2	baculoviral IAP repeat containing 2 [Source:HGNC Symbol;Acc:HGNC:590]
ENSG00000230456	780	DDR1	discoidin domain receptor tyrosine kinase 1 [Source:HGNC Symbol;Acc:HGNC:2730]
ENSG00000263528	9641	IKBKE	inhibitor of kappa light polypeptide gene enhancer in B-cells, kinase epsilon [Source:HGNC Symbol;Acc:HGNC:14552]
ENSG00000140961	29948	OSGIN1	oxidative stress induced growth inhibitor 1 [Source:HGNC Symbol;Acc:HGNC:30093]
ENSG00000173402	1605	DAG1	dystroglycan 1 (dystrophin-associated glycoprotein 1) [Source:HGNC Symbol;Acc:HGNC:2666]
ENSG00000089685	332	BIRC5	baculoviral IAP repeat containing 5 [Source:HGNC Symbol;Acc:HGNC:593]
ENSG00000163638	56999	ADAMTS9	ADAM metallopeptidase with thrombospondin type 1 motif, 9 [Source:HGNC Symbol;Acc:HGNC:13202]
ENSG00000138794	839	CASP6	caspase 6, apoptosis-related cysteine peptidase [Source:HGNC Symbol;Acc:HGNC:1507]
ENSG00000029559	3381	IBSP	integrin-binding sialoprotein [Source:HGNC Symbol;Acc:HGNC:5341]
ENSG00000169436	169044	COL22A1	collagen, type XXII, alpha 1 [Source:HGNC Symbol;Acc:HGNC:22989]
ENSG00000087116	9509	ADAMTS2	ADAM metallopeptidase with thrombospondin type 1 motif, 2 [Source:HGNC Symbol;Acc:HGNC:218]
ENSG00000142192	351	APP	amyloid beta (A4) precursor protein [Source:HGNC Symbol;Acc:HGNC:620]

ENSG00000162552	54361	WNT4	wingless-type MMTV integration site family, member 4 [Source:HGNC Symbol;Acc:HGNC:12783]
ENSG00000138316	140766	ADAMTS14	ADAM metallopeptidase with thrombospondin type 1 motif, 14 [Source:HGNC Symbol;Acc:HGNC:14899]
ENSG00000156709	9131	AIFM1	apoptosis-inducing factor, mitochondrion-associated, 1 [Source:HGNC Symbol;Acc:HGNC:8768]
ENSG00000158874	336	APOA2	apolipoprotein A-II [Source:HGNC Symbol;Acc:HGNC:601]
ENSG00000069702	7049	TGFBR3	transforming growth factor, beta receptor III [Source:HGNC Symbol;Acc:HGNC:11774]
ENSG00000137752	834	CASP1	caspase 1, apoptosis-related cysteine peptidase [Source:HGNC Symbol;Acc:HGNC:1499]
ENSG00000177556	475	ATOX1	antioxidant 1 copper chaperone [Source:HGNC Symbol;Acc:HGNC:798]
ENSG00000154734	9510	ADAMTS1	ADAM metallopeptidase with thrombospondin type 1 motif, 1 [Source:HGNC Symbol;Acc:HGNC:217]
ENSG00000117020	10000	AKT3	v-akt murine thymoma viral oncogene homolog 3 [Source:HGNC Symbol;Acc:HGNC:393]
ENSG00000005884	3675	ITGA3	integrin, alpha 3 (antigen CD49C, alpha 3 subunit of VLA-3 receptor) [Source:HGNC Symbol;Acc:HGNC:6139]
ENSG00000102265	7076	TIMP1	TIMP metallopeptidase inhibitor 1 [Source:HGNC Symbol;Acc:HGNC:11820]
ENSG00000198431	7296	TXNRD1	thioredoxin reductase 1 [Source:HGNC Symbol;Acc:HGNC:12437]
ENSG00000141753	3487	IGFBP4	insulin-like growth factor binding protein 4 [Source:HGNC Symbol;Acc:HGNC:5473]
ENSG00000159267	3141	HLCS	holocarboxylase synthetase (biotin-(proprionyl-CoA-carboxylase (ATP-hydrolysing)) ligase) [Source:HGNC Symbol;Acc:HGNC:4976]
ENSG00000005961	3674	ITGA2B	integrin, alpha 2b (platelet glycoprotein IIb of IIb/IIIa complex, antigen CD41) [Source:HGNC Symbol;Acc:HGNC:6138]
ENSG00000106144	835	CASP2	caspase 2, apoptosis-related cysteine peptidase [Source:HGNC Symbol;Acc:HGNC:1503]
ENSG00000144810	1295	COL8A1	collagen, type VIII, alpha 1 [Source:HGNC Symbol;Acc:HGNC:2215]
ENSG00000223699	1302	COL11A2	collagen, type XI, alpha 2 [Source:HGNC Symbol;Acc:HGNC:2187]
ENSG00000198561	1500	CTNND1	catenin (cadherin-associated protein), delta 1 [Source:HGNC Symbol;Acc:HGNC:2515]

ENSG00000225429	3116	HLA-DPB2	major histocompatibility complex, class II, DP beta 2 (pseudogene) [Source:HGNC Symbol;Acc:HGNC:4941]
ENSG00000269335	8517	IKBKG	inhibitor of kappa light polypeptide gene enhancer in B-cells, kinase gamma [Source:HGNC Symbol;Acc:HGNC:5961]
ENSG00000101981	2158	F9	coagulation factor IX [Source:HGNC Symbol;Acc:HGNC:3551]
ENSG00000151651	101	ADAM8	ADAM metallopeptidase domain 8 [Source:HGNC Symbol;Acc:HGNC:215]
ENSG00000142156	1291	COL6A1	collagen, type VI, alpha 1 [Source:HGNC Symbol;Acc:HGNC:2211]
ENSG00000169598	1677	DFFB	DNA fragmentation factor, 40kDa, beta polypeptide (caspase-activated DNase) [Source:HGNC Symbol;Acc:HGNC:2773]
ENSG00000131174	1349	COX7B	cytochrome c oxidase subunit VIIb [Source:HGNC Symbol;Acc:HGNC:2291]
ENSG00000133433	653689	GSTT2B	glutathione S-transferase theta 2B (gene/pseudogene) [Source:HGNC Symbol;Acc:HGNC:33437]
ENSG00000169814	686	BTD	biotinidase [Source:HGNC Symbol;Acc:HGNC:1122]
ENSG00000164093	5308	PITX2	paired-like homeodomain 2 [Source:HGNC Symbol;Acc:HGNC:9005]
ENSG00000099984	2953	GSTT2	glutathione S-transferase theta 2 (gene/pseudogene) [Source:HGNC Symbol;Acc:HGNC:4642]
ENSG00000215612	3166	HMX1	H6 family homeobox 1 [Source:HGNC Symbol;Acc:HGNC:5017]
ENSG00000122863	9469	CHST3	carbohydrate (chondroitin 6) sulfotransferase 3 [Source:HGNC Symbol;Acc:HGNC:1971]
ENSG00000184216	3654	IRAK1	interleukin-1 receptor-associated kinase 1 [Source:HGNC Symbol;Acc:HGNC:6112]
ENSG00000165240	538	ATP7A	ATPase, Cu++ transporting, alpha polypeptide [Source:HGNC Symbol;Acc:HGNC:869]
ENSG00000077943	8516	ITGA8	integrin, alpha 8 [Source:HGNC Symbol;Acc:HGNC:6144]
ENSG00000171564	2244	FGB	fibrinogen beta chain [Source:HGNC Symbol;Acc:HGNC:3662]
ENSG00000148180	2934	GSN	gelsolin [Source:HGNC Symbol;Acc:HGNC:4620]
ENSG00000182492	633	BGN	biglycan [Source:HGNC Symbol;Acc:HGNC:1044]
ENSG00000115594	3554	IL1R1	interleukin 1 receptor, type I [Source:HGNC Symbol;Acc:HGNC:5993]
ENSG00000134070	3656	IRAK2	interleukin-1 receptor-associated kinase 2 [Source:HGNC Symbol;Acc:HGNC:6113]

ENSG00000213398	3931	LCAT	lecithin-cholesterol acyltransferase [Source:HGNC Symbol;Acc:HGNC:6522]
ENSG00000011465	1634	DCN	decorin [Source:HGNC Symbol;Acc:HGNC:2705]
ENSG00000049540	2006	ELN	elastin [Source:HGNC Symbol;Acc:HGNC:3327]
ENSG00000148400	4851	NOTCH1	notch 1 [Source:HGNC Symbol;Acc:HGNC:7881]
ENSG00000044115	1495	CTNNA1	catenin (cadherin-associated protein), alpha 1, 102kDa [Source:HGNC Symbol;Acc:HGNC:2509]
ENSG00000206290	1302	COL11A2	collagen, type XI, alpha 2 [Source:HGNC Symbol;Acc:HGNC:2187]
ENSG00000104687	2936	GSR	glutathione reductase [Source:HGNC Symbol;Acc:HGNC:4623]
ENSG00000124491	2162	F13A1	coagulation factor XIII, A1 polypeptide [Source:HGNC Symbol;Acc:HGNC:3531]
ENSG00000204262	1290	COL5A2	collagen, type V, alpha 2 [Source:HGNC Symbol;Acc:HGNC:2210]
ENSG00000109610	6649	SOD3	superoxide dismutase 3, extracellular [Source:HGNC Symbol;Acc:HGNC:11181]
ENSG00000163464	3577	CXCR1	chemokine (C-X-C motif) receptor 1 [Source:HGNC Symbol;Acc:HGNC:6026]
ENSG00000001084	2729	GCLC	glutamate-cysteine ligase, catalytic subunit [Source:HGNC Symbol;Acc:HGNC:4311]
ENSG00000186442	3850	KRT3	keratin 3, type II [Source:HGNC Symbol;Acc:HGNC:6440]
ENSG00000130635	1289	COL5A1	collagen, type V, alpha 1 [Source:HGNC Symbol;Acc:HGNC:2209]
ENSG00000183691	9241	NOG	noggin [Source:HGNC Symbol;Acc:HGNC:7866]
ENSG00000125363	265	AMELX	amelogenin, X-linked [Source:HGNC Symbol;Acc:HGNC:461]
ENSG00000204580	780	DDR1	discoidin domain receptor tyrosine kinase 1 [Source:HGNC Symbol;Acc:HGNC:2730]
ENSG00000165029	19	ABCA1	ATP-binding cassette, sub-family A (ABC1), member 1 [Source:HGNC Symbol;Acc:HGNC:29]
ENSG00000277897	2953	GSTT2	glutathione S-transferase theta 2 (gene/pseudogene) [Source:HGNC Symbol;Acc:HGNC:4642]
ENSG00000165272	360	AQP3	aquaporin 3 (Gill blood group) [Source:HGNC Symbol;Acc:HGNC:636]
ENSG00000209082		MT-TL1	mitochondrially encoded tRNA leucine 1 (UUA/G) [Source:HGNC Symbol;Acc:HGNC:7490]
ENSG00000198763	4536	MT-ND2	mitochondrially encoded NADH dehydrogenase 2 [Source:HGNC Symbol;Acc:HGNC:7456]

ENSG00000188130	6300	MAPK12	mitogen-activated protein kinase 12 [Source:HGNC Symbol;Acc:HGNC:6874]
ENSG00000164344	3818	KLKB1	kallikrein B, plasma (Fletcher factor) 1 [Source:HGNC Symbol;Acc:HGNC:6371]
ENSG00000188386	5535	PPP3R2	protein phosphatase 3, regulatory subunit B, beta [Source:HGNC Symbol;Acc:HGNC:9318]
ENSG00000235696	717	C2	complement component 2 [Source:HGNC Symbol;Acc:HGNC:1248]
ENSG00000172156	6356	CCL11	chemokine (C-C motif) ligand 11 [Source:HGNC Symbol;Acc:HGNC:10610]
ENSG00000164924	7534	YWHAZ	tyrosine 3-monooxygenase/tryptophan 5-monooxygenase activation protein, zeta [Source:HGNC Symbol;Acc:HGNC:12855]
ENSG00000008516	64386	MMP25	matrix metallopeptidase 25 [Source:HGNC Symbol;Acc:HGNC:14246]
ENSG00000198691	24	ABCA4	ATP-binding cassette, sub-family A (ABC1), member 4 [Source:HGNC Symbol;Acc:HGNC:34]
ENSG00000143341	83872	HMCN1	hemicentin 1 [Source:HGNC Symbol;Acc:HGNC:19194]
ENSG00000091136	3912	LAMB1	laminin, beta 1 [Source:HGNC Symbol;Acc:HGNC:6486]
ENSG00000138398	9360	PPIG	peptidylprolyl isomerase G (cyclophilin G) [Source:HGNC Symbol;Acc:HGNC:14650]
ENSG00000069956	5597	MAPK6	mitogen-activated protein kinase 6 [Source:HGNC Symbol;Acc:HGNC:6879]
ENSG00000100348	25828	TXN2	thioredoxin 2 [Source:HGNC Symbol;Acc:HGNC:17772]
ENSG00000269741		KLK9	kallikrein-related peptidase 9 [Source:HGNC Symbol;Acc:HGNC:6370]
ENSG00000167757	11012	KLK11	kallikrein-related peptidase 11 [Source:HGNC Symbol;Acc:HGNC:6359]
ENSG00000118785	6696	SPP1	secreted phosphoprotein 1 [Source:HGNC Symbol;Acc:HGNC:11255]
ENSG00000167755	5653	KLK6	kallikrein-related peptidase 6 [Source:HGNC Symbol;Acc:HGNC:6367]
ENSG00000147065	4478	MSN	moesin [Source:HGNC Symbol;Acc:HGNC:7373]
ENSG00000092200	57096	RPGRIP1	retinitis pigmentosa GTPase regulator interacting protein 1 [Source:HGNC Symbol;Acc:HGNC:13436]
ENSG00000081148	50939	IMPG2	interphotoreceptor matrix proteoglycan 2 [Source:HGNC Symbol;Acc:HGNC:18362]
ENSG00000117676	6195	RPS6KA1	ribosomal protein S6 kinase, 90kDa, polypeptide 1 [Source:HGNC Symbol;Acc:HGNC:10430]
ENSG00000112769	3910	LAMA4	laminin, alpha 4 [Source:HGNC Symbol;Acc:HGNC:6484]

ENSG00000087303	22795	NID2	nidogen 2 (osteonidogen) [Source:HGNC Symbol;Acc:HGNC:13389]
ENSG00000164867	4846	NOS3	nitric oxide synthase 3 (endothelial cell) [Source:HGNC Symbol;Acc:HGNC:7876]
ENSG00000282637	9020	MAP3K14	mitogen-activated protein kinase kinase kinase 14 [Source:HGNC Symbol;Acc:HGNC:6853]
ENSG00000113520	3565	IL4	interleukin 4 [Source:HGNC Symbol;Acc:HGNC:6014]
ENSG00000041982	3371	TNC	tenascin C [Source:HGNC Symbol;Acc:HGNC:5318]
ENSG00000185591	6667	SP1	Sp1 transcription factor [Source:HGNC Symbol;Acc:HGNC:11205]
ENSG00000206439	7124	TNF	tumor necrosis factor [Source:HGNC Symbol;Acc:HGNC:11892]
ENSG00000121634	2703	GJA8	gap junction protein, alpha 8, 50kDa [Source:HGNC Symbol;Acc:HGNC:4281]
ENSG00000244414	3078	CFHR1	complement factor H-related 1 [Source:HGNC Symbol;Acc:HGNC:4888]
ENSG00000136869	7099	TLR4	toll-like receptor 4 [Source:HGNC Symbol;Acc:HGNC:11850]
ENSG00000104368	5327	PLAT	plasminogen activator, tissue [Source:HGNC Symbol;Acc:HGNC:9051]
ENSG00000042781	7399	USH2A	Usher syndrome 2A (autosomal recessive, mild) [Source:HGNC Symbol;Acc:HGNC:12601]
ENSG00000108953	7531	YWHAE	tyrosine 3-monooxygenase/tryptophan 5-monooxygenase activation protein, epsilon [Source:HGNC Symbol;Acc:HGNC:12851]
ENSG00000104497	64089	SNX16	sorting nexin 16 [Source:HGNC Symbol;Acc:HGNC:14980]
ENSG00000151276	9223	MAGI1	membrane associated guanylate kinase, WW and PDZ domain containing 1 [Source:HGNC Symbol;Acc:HGNC:946]
ENSG00000102871	8717	TRADD	TNFRSF1A-associated via death domain [Source:HGNC Symbol;Acc:HGNC:12030]
ENSG00000122482	84146	ZNF644	zinc finger protein 644 [Source:HGNC Symbol;Acc:HGNC:29222]
ENSG00000114302	5576	PRKAR2A	protein kinase, cAMP-dependent, regulatory, type II, alpha [Source:HGNC Symbol;Acc:HGNC:9391]
ENSG00000102878	3299	HSF4	heat shock transcription factor 4 [Source:HGNC Symbol;Acc:HGNC:5227]
ENSG00000137673	4316	MMP7	matrix metallopeptidase 7 [Source:HGNC Symbol;Acc:HGNC:7174]
ENSG00000119614	338917	VSX2	visual system homeobox 2 [Source:HGNC Symbol;Acc:HGNC:1975]
ENSG00000154485	118856	MMP21	matrix metallopeptidase 21 [Source:HGNC Symbol;Acc:HGNC:14357]
ENSG00000129455	11202	KLK8	kallikrein-related peptidase 8 [Source:HGNC Symbol;Acc:HGNC:6369]

ENSG00000110799	7450	VWF	von Willebrand factor [Source:HGNC Symbol;Acc:HGNC:12726]
ENSG00000126226	55795	PCID2	PCI domain containing 2 [Source:HGNC Symbol;Acc:HGNC:25653]
ENSG00000130489	9997	SCO2	SCO2 cytochrome c oxidase assembly protein [Source:HGNC Symbol;Acc:HGNC:10604]
ENSG00000115221	3694	ITGB6	integrin, beta 6 [Source:HGNC Symbol;Acc:HGNC:6161]
ENSG00000169035	5650	KLK7	kallikrein-related peptidase 7 [Source:HGNC Symbol;Acc:HGNC:6368]
ENSG00000271447	79148	MMP28	matrix metallopeptidase 28 [Source:HGNC Symbol;Acc:HGNC:14366]
ENSG00000172236	7177	TPSAB1	tryptase alpha/beta 1 [Source:HGNC Symbol;Acc:HGNC:12019]
ENSG00000099953	4320	MMP11	matrix metallopeptidase 11 [Source:HGNC Symbol;Acc:HGNC:7157]
ENSG00000112486	1235	CCR6	chemokine (C-C motif) receptor 6 [Source:HGNC Symbol;Acc:HGNC:1607]
ENSG00000196811	1146	CHRNG	cholinergic receptor, nicotinic, gamma (muscle) [Source:HGNC Symbol;Acc:HGNC:1967]
ENSG00000198933	9755	TBKBP1	TBK1 binding protein 1 [Source:HGNC Symbol;Acc:HGNC:30140]
ENSG00000282307	5176	SERPINF1	serpin peptidase inhibitor, clade F (alpha-2 antiplasmin, pigment epithelium derived factor), member 1 [Source:HGNC Symbol;Acc:HGNC:8824]
ENSG00000178726	7056	THBD	thrombomodulin [Source:HGNC Symbol;Acc:HGNC:11784]
ENSG00000274247	5644	PRSS1	protease, serine, 1 (trypsin 1) [Source:HGNC Symbol;Acc:HGNC:9475]
ENSG00000100241	6305	SBF1	SET binding factor 1 [Source:HGNC Symbol;Acc:HGNC:10542]
ENSG00000166913	7529	YWHAB	tyrosine 3-monooxygenase/tryptophan 5-monooxygenase activation protein, beta [Source:HGNC Symbol;Acc:HGNC:12849]
ENSG00000167754	25818	KLK5	kallikrein-related peptidase 5 [Source:HGNC Symbol;Acc:HGNC:6366]
ENSG00000188937	60506	NYX	nyctalopin [Source:HGNC Symbol;Acc:HGNC:8082]
ENSG00000101938	91851	CHRDL1	chordin-like 1 [Source:HGNC Symbol;Acc:HGNC:29861]
ENSG00000100207	6942	TCF20	transcription factor 20 (AR1) [Source:HGNC Symbol;Acc:HGNC:11631]
ENSG00000048545	2978	GUCA1A	guanylate cyclase activator 1A (retina) [Source:HGNC Symbol;Acc:HGNC:4678]
ENSG00000214140	768206	PRCD	progressive rod-cone degeneration [Source:HGNC Symbol;Acc:HGNC:32528]
ENSG00000188452	375298	CERKL	ceramide kinase-like [Source:HGNC Symbol;Acc:HGNC:21699]

ENSG00000113140	6678	SPARC	secreted protein, acidic, cysteine-rich (osteonectin) [Source:HGNC Symbol;Acc:HGNC:11219]
ENSG00000118271	7276	TTR	transthyretin [Source:HGNC Symbol;Acc:HGNC:12405]
ENSG00000138814	5530	PPP3CA	protein phosphatase 3, catalytic subunit, alpha isozyme [Source:HGNC Symbol;Acc:HGNC:9314]
ENSG00000136381	3658	IREB2	iron-responsive element binding protein 2 [Source:HGNC Symbol;Acc:HGNC:6115]
ENSG00000172037	3913	LAMB2	laminin, beta 2 (laminin S) [Source:HGNC Symbol;Acc:HGNC:6487]
ENSG00000137674	9313	MMP20	matrix metallopeptidase 20 [Source:HGNC Symbol;Acc:HGNC:7167]
ENSG00000068078	2261	FGFR3	fibroblast growth factor receptor 3 [Source:HGNC Symbol;Acc:HGNC:3690]
ENSG00000087053	8898	MTMR2	myotubularin related protein 2 [Source:HGNC Symbol;Acc:HGNC:7450]
ENSG00000137675	64066	MMP27	matrix metallopeptidase 27 [Source:HGNC Symbol;Acc:HGNC:14250]
ENSG00000115318	84695	LOXL3	lysyl oxidase-like 3 [Source:HGNC Symbol;Acc:HGNC:13869]
ENSG00000122861	5328	PLAU	plasminogen activator, urokinase [Source:HGNC Symbol;Acc:HGNC:9052]
ENSG00000101680	284217	LAMA1	laminin, alpha 1 [Source:HGNC Symbol;Acc:HGNC:6481]
ENSG00000128245	7533	YWHAH	tyrosine 3-monooxygenase/tryptophan 5-monooxygenase activation protein, eta [Source:HGNC Symbol;Acc:HGNC:12853]
ENSG00000198363	444	ASPH	aspartate beta-hydroxylase [Source:HGNC Symbol;Acc:HGNC:757]
ENSG00000119699	7043	TGFB3	transforming growth factor, beta 3 [Source:HGNC Symbol;Acc:HGNC:11769]
ENSG00000105825	7980	TFPI2	tissue factor pathway inhibitor 2 [Source:HGNC Symbol;Acc:HGNC:11761]
ENSG00000130164	3949	LDLR	low density lipoprotein receptor [Source:HGNC Symbol;Acc:HGNC:6547]
ENSG00000136810	7295	TXN	thioredoxin [Source:HGNC Symbol;Acc:HGNC:12435]
ENSG00000162692	7412	VCAM1	vascular cell adhesion molecule 1 [Source:HGNC Symbol;Acc:HGNC:12663]
ENSG00000121858	8743	TNFSF10	tumor necrosis factor (ligand) superfamily, member 10 [Source:HGNC Symbol;Acc:HGNC:11925]
ENSG00000086991	50507	NOX4	NADPH oxidase 4 [Source:HGNC Symbol;Acc:HGNC:7891]
ENSG00000184302	4990	SIX6	SIX homeobox 6 [Source:HGNC Symbol;Acc:HGNC:10892]
ENSG00000138755	4283	CXCL9	chemokine (C-X-C motif) ligand 9 [Source:HGNC Symbol;Acc:HGNC:7098]

ENSG00000170289	54714	CNGB3	cyclic nucleotide gated channel beta 3 [Source:HGNC Symbol;Acc:HGNC:2153]
ENSG00000186474	43849	KLK12	kallikrein-related peptidase 12 [Source:HGNC Symbol;Acc:HGNC:6360]
ENSG00000231408	4049	LTA	lymphotoxin alpha [Source:HGNC Symbol;Acc:HGNC:6709]
ENSG00000102962	6367	CCL22	chemokine (C-C motif) ligand 22 [Source:HGNC Symbol;Acc:HGNC:10621]
ENSG00000010704	3077	HFE	hemochromatosis [Source:HGNC Symbol;Acc:HGNC:4886]
ENSG00000078401	1906	EDN1	endothelin 1 [Source:HGNC Symbol;Acc:HGNC:3176]
ENSG00000156711	5603	MAPK13	mitogen-activated protein kinase 13 [Source:HGNC Symbol;Acc:HGNC:6875]
ENSG00000107758	5532	PPP3CB	protein phosphatase 3, catalytic subunit, beta isozyme [Source:HGNC Symbol;Acc:HGNC:9315]
ENSG00000276461	6942	TCF20	transcription factor 20 (AR1) [Source:HGNC Symbol;Acc:HGNC:11631]
ENSG00000188158	4810	NHS	Nance-Horan syndrome (congenital cataracts and dental anomalies) [Source:HGNC Symbol;Acc:HGNC:7820]
ENSG00000215914		MMP23A	matrix metallopeptidase 23A (pseudogene) [Source:HGNC Symbol;Acc:HGNC:7170]
ENSG00000149489	6094	ROM1	retinal outer segment membrane protein 1 [Source:HGNC Symbol;Acc:HGNC:10254]
ENSG00000119681	4053	LTBP2	latent transforming growth factor beta binding protein 2 [Source:HGNC Symbol;Acc:HGNC:6715]
ENSG00000189409	8510	MMP23B	matrix metallopeptidase 23B [Source:HGNC Symbol;Acc:HGNC:7171]
ENSG00000156103	4325	MMP16	matrix metallopeptidase 16 (membrane-inserted) [Source:HGNC Symbol;Acc:HGNC:7162]
ENSG00000163956	4043	LRPAP1	low density lipoprotein receptor-related protein associated protein 1 [Source:HGNC Symbol;Acc:HGNC:6701]
ENSG00000166794	5479	PPIB	peptidylprolyl isomerase B (cyclophilin B) [Source:HGNC Symbol;Acc:HGNC:9255]
ENSG00000196611	4312	MMP1	matrix metallopeptidase 1 [Source:HGNC Symbol;Acc:HGNC:7155]
ENSG00000168078	55872	PBK	PDZ binding kinase [Source:HGNC Symbol;Acc:HGNC:18282]
ENSG00000104921	2208	FCER2	Fc fragment of IgE, low affinity II, receptor for (CD23) [Source:HGNC Symbol;Acc:HGNC:3612]
ENSG00000167165	54578	UGT1A6	UDP glucuronosyltransferase 1 family, polypeptide A6 [Source:HGNC Symbol;Acc:HGNC:12538]

ENSG00000149968	4314	MMP3	matrix metallopeptidase 3 [Source:HGNC Symbol;Acc:HGNC:7173]
ENSG00000173535	8794	TNFRSF10C	tumor necrosis factor receptor superfamily, member 10c, decoy without an intracellular domain [Source:HGNC Symbol;Acc:HGNC:11906]
ENSG00000030304	4593	MUSK	muscle, skeletal, receptor tyrosine kinase [Source:HGNC Symbol;Acc:HGNC:7525]
ENSG00000230279	4049	LTA	lymphotoxin alpha [Source:HGNC Symbol;Acc:HGNC:6709]
ENSG00000149257	871	SERPINH1	serpin peptidase inhibitor, clade H (heat shock protein 47), member 1, (collagen binding protein 1) [Source:HGNC Symbol;Acc:HGNC:1546]
ENSG00000238130	4049	LTA	lymphotoxin alpha [Source:HGNC Symbol;Acc:HGNC:6709]
ENSG00000134987	134430	WDR36	WD repeat domain 36 [Source:HGNC Symbol;Acc:HGNC:30696]
ENSG00000105370	3982	LIM2	lens intrinsic membrane protein 2, 19kDa [Source:HGNC Symbol;Acc:HGNC:6610]
ENSG00000152785	651	BMP3	bone morphogenetic protein 3 [Source:HGNC Symbol;Acc:HGNC:1070]
ENSG00000109103	9094	UNC119	unc-119 homolog (C. elegans) [Source:HGNC Symbol;Acc:HGNC:12565]
ENSG00000117461	8503	PIK3R3	phosphoinositide-3-kinase, regulatory subunit 3 (gamma) [Source:HGNC Symbol;Acc:HGNC:8981]
ENSG00000115884	6382	SDC1	syndecan 1 [Source:HGNC Symbol;Acc:HGNC:10658]
ENSG00000204364	717	C2	complement component 2 [Source:HGNC Symbol;Acc:HGNC:1248]
ENSG00000135517	4284	MIP	major intrinsic protein of lens fiber [Source:HGNC Symbol;Acc:HGNC:7103]
ENSG00000134569	4038	LRP4	low density lipoprotein receptor-related protein 4 [Source:HGNC Symbol;Acc:HGNC:6696]
ENSG00000142627	1969	EPHA2	EPH receptor A2 [Source:HGNC Symbol;Acc:HGNC:3386]
ENSG00000104237	6101	RP1	retinitis pigmentosa 1 (autosomal dominant) [Source:HGNC Symbol;Acc:HGNC:10263]
ENSG00000173530	8793	TNFRSF10D	tumor necrosis factor receptor superfamily, member 10d, decoy with truncated death domain [Source:HGNC Symbol;Acc:HGNC:11907]
ENSG00000278139	8503	PIK3R3	phosphoinositide-3-kinase, regulatory subunit 3 (gamma) [Source:EntrezGene;Acc:8503]
ENSG00000038295	7092	TLL1	tolloid-like 1 [Source:HGNC Symbol;Acc:HGNC:11843]

ENSG00000228321	7124	TNF	tumor necrosis factor [Source:HGNC Symbol;Acc:HGNC:11892]
ENSG00000112041	7287	TULP1	tubby like protein 1 [Source:HGNC Symbol;Acc:HGNC:12423]
ENSG00000039139	1767	DNAH5	dynein, axonemal, heavy chain 5 [Source:HGNC Symbol;Acc:HGNC:2950]
ENSG00000121743	2700	GJA3	gap junction protein, alpha 3, 46kDa [Source:HGNC Symbol;Acc:HGNC:4277]
ENSG00000123240	10133	OPTN	optineurin [Source:HGNC Symbol;Acc:HGNC:17142]
ENSG00000082781	3693	ITGB5	integrin, beta 5 [Source:HGNC Symbol;Acc:HGNC:6160]
ENSG00000228978	7124	TNF	tumor necrosis factor [Source:HGNC Symbol;Acc:HGNC:11892]
ENSG00000237412	646960	PRSS56	protease, serine, 56 [Source:HGNC Symbol;Acc:HGNC:39433]
ENSG00000173801	3728	JUP	junction plakoglobin [Source:HGNC Symbol;Acc:HGNC:6207]
ENSG00000054598	2296	FOXC1	forkhead box C1 [Source:HGNC Symbol;Acc:HGNC:3800]
ENSG00000231608	7148	TNXB	tenascin XB [Source:HGNC Symbol;Acc:HGNC:11976]
ENSG00000226275	4049	LTA	lymphotoxin alpha [Source:HGNC Symbol;Acc:HGNC:6709]
ENSG00000102970	6361	CCL17	chemokine (C-C motif) ligand 17 [Source:HGNC Symbol;Acc:HGNC:10615]
ENSG00000168958	56947	MFF	mitochondrial fission factor [Source:HGNC Symbol;Acc:HGNC:24858]
ENSG00000083444	5351	PLOD1	procollagen-lysine, 2-oxoglutarate 5-dioxygenase 1 [Source:HGNC Symbol;Acc:HGNC:9081]
ENSG00000167759	26085	KLK13	kallikrein-related peptidase 13 [Source:HGNC Symbol;Acc:HGNC:6361]
ENSG00000105392	1406	CRX	cone-rod homeobox [Source:HGNC Symbol;Acc:HGNC:2383]
ENSG00000104689	8797	TNFRSF10A	tumor necrosis factor receptor superfamily, member 10a [Source:HGNC Symbol;Acc:HGNC:11904]
ENSG00000139053	5149	PDE6H	phosphodiesterase 6H, cGMP-specific, cone, gamma [Source:HGNC Symbol;Acc:HGNC:8790]
ENSG00000087245	4313	MMP2	matrix metallopeptidase 2 [Source:HGNC Symbol;Acc:HGNC:7166]
ENSG00000204359	629	CFB	complement factor B [Source:HGNC Symbol;Acc:HGNC:1037]
ENSG00000167346	56547	MMP26	matrix metallopeptidase 26 [Source:HGNC Symbol;Acc:HGNC:14249]
ENSG00000235017	717	C2	complement component 2 [Source:HGNC Symbol;Acc:HGNC:1248]
ENSG00000196569	3908	LAMA2	laminin, alpha 2 [Source:HGNC Symbol;Acc:HGNC:6482]

ENSG00000139988	145226	RDH12	retinol dehydrogenase 12 (all-trans/9-cis/11-cis) [Source:HGNC Symbol;Acc:HGNC:19977]
ENSG00000144028	23020	SNRNP200	small nuclear ribonucleoprotein 200kDa (U5) [Source:HGNC Symbol;Acc:HGNC:30859]
ENSG00000120889	8795	TNFRSF10B	tumor necrosis factor receptor superfamily, member 10b [Source:HGNC Symbol;Acc:HGNC:11905]
ENSG00000109320	4790	NFKB1	nuclear factor of kappa light polypeptide gene enhancer in B-cells 1 [Source:HGNC Symbol;Acc:HGNC:7794]
ENSG00000243649	629	CFB	complement factor B [Source:HGNC Symbol;Acc:HGNC:1037]
ENSG00000100311	5155	PDGFB	platelet-derived growth factor beta polypeptide [Source:HGNC Symbol;Acc:HGNC:8800]
ENSG00000171608	5293	PIK3CD	phosphatidylinositol-4,5-bisphosphate 3-kinase, catalytic subunit delta [Source:HGNC Symbol;Acc:HGNC:8977]
ENSG00000141506	23533	PIK3R5	phosphoinositide-3-kinase, regulatory subunit 5 [Source:HGNC Symbol;Acc:HGNC:30035]
ENSG00000162510	4146	MATN1	matrilin 1, cartilage matrix protein [Source:HGNC Symbol;Acc:HGNC:6907]
ENSG00000133812	81846	SBF2	SET binding factor 2 [Source:HGNC Symbol;Acc:HGNC:2135]
ENSG00000116745	6121	RPE65	retinal pigment epithelium-specific protein 65kDa [Source:HGNC Symbol;Acc:HGNC:10294]
ENSG00000112715	7422	VEGFA	vascular endothelial growth factor A [Source:HGNC Symbol;Acc:HGNC:12680]
ENSG00000129437	43847	KLK14	kallikrein-related peptidase 14 [Source:HGNC Symbol;Acc:HGNC:6362]
ENSG00000156313	6103	RPGR	retinitis pigmentosa GTPase regulator [Source:HGNC Symbol;Acc:HGNC:10295]
ENSG00000108946	5573	PRKAR1A	protein kinase, cAMP-dependent, regulatory, type I, alpha [Source:HGNC Symbol;Acc:HGNC:9388]
ENSG00000087086	2512	FTL	ferritin, light polypeptide [Source:HGNC Symbol;Acc:HGNC:3999]
ENSG00000225950	4909	NTF4	neurotrophin 4 [Source:HGNC Symbol;Acc:HGNC:8024]
ENSG00000134184	2944	GSTM1	glutathione S-transferase mu 1 [Source:HGNC Symbol;Acc:HGNC:4632]
ENSG00000165474	2706	GJB2	gap junction protein, beta 2, 26kDa [Source:HGNC Symbol;Acc:HGNC:4284]
ENSG00000112062	1432	MAPK14	mitogen-activated protein kinase 14 [Source:HGNC Symbol;Acc:HGNC:6876]
ENSG00000122550	55975	KLHL7	kelch-like family member 7 [Source:HGNC Symbol;Acc:HGNC:15646]

ENSG00000122194	5340	PLG	plasminogen [Source:HGNC Symbol;Acc:HGNC:9071]
ENSG00000160202	1409	CRYAA	crystallin, alpha A [Source:HGNC Symbol;Acc:HGNC:2388]
ENSG00000226979	4049	LTA	lymphotoxin alpha [Source:HGNC Symbol;Acc:HGNC:6709]
ENSG00000112115	3605	IL17A	interleukin 17A [Source:HGNC Symbol;Acc:HGNC:5981]
ENSG00000095596	1592	CYP26A1	cytochrome P450, family 26, subfamily A, polypeptide 1 [Source:HGNC Symbol;Acc:HGNC:2603]
ENSG00000152952	5352	PLOD2	procollagen-lysine, 2-oxoglutarate 5-dioxygenase 2 [Source:HGNC Symbol;Acc:HGNC:9082]
ENSG00000163931	7086	TKT	transketolase [Source:HGNC Symbol;Acc:HGNC:11834]
ENSG00000134183	2780	GNAT2	guanine nucleotide binding protein (G protein), alpha transducing activity polypeptide 2 [Source:HGNC Symbol;Acc:HGNC:4394]
ENSG00000239754	629	CFB	complement factor B [Source:HGNC Symbol;Acc:HGNC:1037]
ENSG00000185386	5600	MAPK11	mitogen-activated protein kinase 11 [Source:HGNC Symbol;Acc:HGNC:6873]
ENSG00000100225	25793	FBXO7	F-box protein 7 [Source:HGNC Symbol;Acc:HGNC:13586]
ENSG00000223952	7124	TNF	tumor necrosis factor [Source:HGNC Symbol;Acc:HGNC:11892]
ENSG00000261371	5175	PECAM1	platelet/endothelial cell adhesion molecule 1 [Source:HGNC Symbol;Acc:HGNC:8823]
ENSG00000108688	6354	CCL7	chemokine (C-C motif) ligand 7 [Source:HGNC Symbol;Acc:HGNC:10634]
ENSG00000051382	5291	PIK3CB	phosphatidylinositol-4,5-bisphosphate 3-kinase, catalytic subunit beta [Source:HGNC Symbol;Acc:HGNC:8976]
ENSG00000135437	5959	RDH5	retinol dehydrogenase 5 (11-cis/9-cis) [Source:HGNC Symbol;Acc:HGNC:9940]
ENSG00000282752	9150	CTDP1	CTD (carboxy-terminal domain, RNA polymerase II, polypeptide A) phosphatase, subunit 1 [Source:HGNC Symbol;Acc:HGNC:2498]
ENSG00000243570	629	CFB	complement factor B [Source:HGNC Symbol;Acc:HGNC:1037]
ENSG00000236236	7148	TNXB	tenascin XB [Source:HGNC Symbol;Acc:HGNC:11976]
ENSG00000169397	6037	RNASE3	ribonuclease, RNase A family, 3 [Source:HGNC Symbol;Acc:HGNC:10046]
ENSG00000138131	84171	LOXL4	lysyl oxidase-like 4 [Source:HGNC Symbol;Acc:HGNC:17171]

ENSG00000173039	5970	RELA	v-rel avian reticuloendotheliosis viral oncogene homolog A [Source:HGNC Symbol;Acc:HGNC:9955]
ENSG00000168056	4054	LTBP3	latent transforming growth factor beta binding protein 3 [Source:HGNC Symbol;Acc:HGNC:6716]
ENSG00000166278	717	C2	complement component 2 [Source:HGNC Symbol;Acc:HGNC:1248]
ENSG00000179915	9378	NRXN1	neurexin 1 [Source:HGNC Symbol;Acc:HGNC:8008]
ENSG00000107643	5599	MAPK8	mitogen-activated protein kinase 8 [Source:HGNC Symbol;Acc:HGNC:6881]
ENSG00000197579	10210	TOPORS	topoisomerase I binding, arginine/serine-rich, E3 ubiquitin protein ligase [Source:HGNC Symbol;Acc:HGNC:21653]
ENSG00000125730	718	C3	complement component 3 [Source:HGNC Symbol;Acc:HGNC:1318]
ENSG00000006530	55750	AGK	acylglycerol kinase [Source:HGNC Symbol;Acc:HGNC:21869]
ENSG00000137275	8737	RIPK1	receptor (TNFRSF)-interacting serine-threonine kinase 1 [Source:HGNC Symbol;Acc:HGNC:10019]
ENSG00000109339	5602	MAPK10	mitogen-activated protein kinase 10 [Source:HGNC Symbol;Acc:HGNC:6872]
ENSG00000196655	51399	TRAPPC4	trafficking protein particle complex 4 [Source:HGNC Symbol;Acc:HGNC:19943]
ENSG00000125864	631	BFSP1	beaded filament structural protein 1, filensin [Source:HGNC Symbol;Acc:HGNC:1040]
ENSG00000164007	149461	CLDN19	claudin 19 [Source:HGNC Symbol;Acc:HGNC:2040]
ENSG00000170421	3856	KRT8	keratin 8, type II [Source:HGNC Symbol;Acc:HGNC:6446]
ENSG00000143183	54499	TMCO1	transmembrane and coiled-coil domains 1 [Source:HGNC Symbol;Acc:HGNC:18188]
ENSG00000140092	10516	FBLN5	fibulin 5 [Source:HGNC Symbol;Acc:HGNC:3602]
ENSG00000100053	1417	CRYBB3	crystallin, beta B3 [Source:HGNC Symbol;Acc:HGNC:2400]
ENSG00000120332	63923	TNN	tenascin N [Source:HGNC Symbol;Acc:HGNC:22942]
ENSG00000112033	5467	PPARD	peroxisome proliferator-activated receptor delta [Source:HGNC Symbol;Acc:HGNC:9235]
ENSG00000178573	4094	MAF	v-maf avian musculoaponeurotic fibrosarcoma oncogene homolog [Source:HGNC Symbol;Acc:HGNC:6776]
ENSG00000132470	3691	ITGB4	integrin, beta 4 [Source:HGNC Symbol;Acc:HGNC:6158]

ENSG00000213139	1427	CRYGS	crystallin, gamma S [Source:HGNC Symbol;Acc:HGNC:2417]
ENSG00000113889	3827	KNG1	kininogen 1 [Source:HGNC Symbol;Acc:HGNC:6383]
ENSG00000165059	5568	PRKACG	protein kinase, cAMP-dependent, catalytic, gamma [Source:HGNC Symbol;Acc:HGNC:9382]
ENSG00000102996	4324	MMP15	matrix metallopeptidase 15 (membrane-inserted) [Source:HGNC Symbol;Acc:HGNC:7161]
ENSG00000226560	717	C2	complement component 2 [Source:HGNC Symbol;Acc:HGNC:1248]
ENSG00000034971	4653	MYOC	myocilin, trabecular meshwork inducible glucocorticoid response [Source:HGNC Symbol;Acc:HGNC:7610]
ENSG00000105647	5296	PIK3R2	phosphoinositide-3-kinase, regulatory subunit 2 (beta) [Source:HGNC Symbol;Acc:HGNC:8980]
ENSG00000241253	629	CFB	complement factor B [Source:HGNC Symbol;Acc:HGNC:1037]
ENSG00000112130	9025	RNF8	ring finger protein 8, E3 ubiquitin protein ligase [Source:HGNC Symbol;Acc:HGNC:10071]
ENSG00000169248	6373	CXCL11	chemokine (C-X-C motif) ligand 11 [Source:HGNC Symbol;Acc:HGNC:10638]
ENSG00000130203	348	APOE	apolipoprotein E [Source:HGNC Symbol;Acc:HGNC:613]
ENSG00000268173		PIK3R2	phosphoinositide-3-kinase, regulatory subunit 2 (beta) [Source:HGNC Symbol;Acc:HGNC:8980]
ENSG00000241534	629	CFB	complement factor B [Source:HGNC Symbol;Acc:HGNC:1037]
ENSG00000204490	7124	TNF	tumor necrosis factor [Source:HGNC Symbol;Acc:HGNC:11892]
ENSG00000113083	4015	LOX	lysyl oxidase [Source:HGNC Symbol;Acc:HGNC:6664]
ENSG00000157150	7079	TIMP4	TIMP metallopeptidase inhibitor 4 [Source:HGNC Symbol;Acc:HGNC:11823]
ENSG00000132031	4148	MATN3	matrilin 3 [Source:HGNC Symbol;Acc:HGNC:6909]
ENSG00000105426	5802	PTPRS	protein tyrosine phosphatase, receptor type, S [Source:HGNC Symbol;Acc:HGNC:9681]
ENSG00000169194	3596	IL13	interleukin 13 [Source:HGNC Symbol;Acc:HGNC:5973]
ENSG00000184470	10587	TXNRD2	thioredoxin reductase 2 [Source:HGNC Symbol;Acc:HGNC:18155]
ENSG00000116044	4780	NFE2L2	nuclear factor, erythroid 2-like 2 [Source:HGNC Symbol;Acc:HGNC:7782]
ENSG00000189221	4128	MAOA	monoamine oxidase A [Source:HGNC Symbol;Acc:HGNC:6833]
ENSG00000134308	10971	YWHAQ	tyrosine 3-monooxygenase/tryptophan 5-monooxygenase activation protein [illegible]

ENSG00000262327	55750	AGK	acylglycerol kinase [Source:HGNC Symbol;Acc:HGNC:21869]
ENSG00000163710	26577	PCOLCE2	procollagen C-endopeptidase enhancer 2 [Source:HGNC Symbol;Acc:HGNC:8739]
ENSG00000113525	3567	IL5	interleukin 5 [Source:HGNC Symbol;Acc:HGNC:6016]
ENSG00000079999	9817	KEAP1	kelch-like ECH-associated protein 1 [Source:HGNC Symbol;Acc:HGNC:23177]
ENSG00000123342	4327	MMP19	matrix metallopeptidase 19 [Source:HGNC Symbol;Acc:HGNC:7165]
ENSG00000038427	1462	VCAN	versican [Source:HGNC Symbol;Acc:HGNC:2464]
ENSG00000134259	4803	NGF	nerve growth factor (beta polypeptide) [Source:HGNC Symbol;Acc:HGNC:7808]
ENSG00000137801	7057	THBS1	thrombospondin 1 [Source:HGNC Symbol;Acc:HGNC:11785]
ENSG00000107679	59338	PLEKHA1	pleckstrin homology domain containing, family A (phosphoinositide binding specific) member 1 [Source:HGNC Symbol;Acc:HGNC:14335]
ENSG00000254636	387715	ARMS2	age-related maculopathy susceptibility 2 [Source:HGNC Symbol;Acc:HGNC:32685]
ENSG00000164022	9255	AIMP1	aminoacyl tRNA synthetase complex-interacting multifunctional protein 1 [Source:HGNC Symbol;Acc:HGNC:10648]
ENSG00000187193	4501	MT1X	metallothionein 1X [Source:HGNC Symbol;Acc:HGNC:7405]
ENSG00000188191	5575	PRKAR1B	protein kinase, cAMP-dependent, regulatory, type I, beta [Source:HGNC Symbol;Acc:HGNC:9390]
ENSG00000085662	231	AKR1B1	aldo-keto reductase family 1, member B1 (aldose reductase) [Source:HGNC Symbol;Acc:HGNC:381]
ENSG00000173503	4049	LTA	lymphotoxin alpha [Source:HGNC Symbol;Acc:HGNC:6709]
ENSG00000105851	5294	PIK3CG	phosphatidylinositol-4,5-bisphosphate 3-kinase, catalytic subunit gamma [Source:HGNC Symbol;Acc:HGNC:8978]
ENSG00000242335	629	CFB	complement factor B [Source:HGNC Symbol;Acc:HGNC:1037]
ENSG00000225830	2074	ERCC6	excision repair cross-complementation group 6 [Source:HGNC Symbol;Acc:HGNC:3438]
ENSG00000162512	9672	SDC3	syndecan 3 [Source:HGNC Symbol;Acc:HGNC:10660]
ENSG00000149294	4684	NCAM1	neural cell adhesion molecule 1 [Source:HGNC Symbol;Acc:HGNC:7656]

ENSG00000095587	7093	TLL2	tolloid-like 2 [Source:HGNC Symbol;Acc:HGNC:11844]
ENSG00000233323	7148	TNXB	tenascin XB [Source:HGNC Symbol;Acc:HGNC:11976]
ENSG00000133256	5158	PDE6B	phosphodiesterase 6B, cGMP-specific, rod, beta [Source:HGNC Symbol;Acc:HGNC:8786]
ENSG00000167995	7439	BEST1	bestrophin 1 [Source:HGNC Symbol;Acc:HGNC:12703]
ENSG00000050748	5601	MAPK9	mitogen-activated protein kinase 9 [Source:HGNC Symbol;Acc:HGNC:6886]
ENSG00000185624	5034	P4HB	prolyl 4-hydroxylase, beta polypeptide [Source:HGNC Symbol;Acc:HGNC:8548]
ENSG00000166033	5654	HTRA1	HtrA serine peptidase 1 [Source:HGNC Symbol;Acc:HGNC:9476]
ENSG00000170027	7532	YWHAG	tyrosine 3-monooxygenase/tryptophan 5-monooxygenase activation protein, gamma [Source:HGNC Symbol;Acc:HGNC:12852]
ENSG00000185176	653437	AQP12B	aquaporin 12B [Source:HGNC Symbol;Acc:HGNC:6096]
ENSG00000117586	7292	TNFSF4	tumor necrosis factor (ligand) superfamily, member 4 [Source:HGNC Symbol;Acc:HGNC:11934]
ENSG00000229341	7148	TNXB	tenascin XB [Source:HGNC Symbol;Acc:HGNC:11976]
ENSG00000102218	6102	RP2	retinitis pigmentosa 2 (X-linked recessive) [Source:HGNC Symbol;Acc:HGNC:10274]
ENSG00000198598	4326	MMP17	matrix metallopeptidase 17 (membrane-inserted) [Source:HGNC Symbol;Acc:HGNC:7163]
ENSG00000142875	5567	PRKACB	protein kinase, cAMP-dependent, catalytic, beta [Source:HGNC Symbol;Acc:HGNC:9381]
ENSG00000116830	8458	TTF2	transcription termination factor, RNA polymerase II [Source:HGNC Symbol;Acc:HGNC:12398]
ENSG00000116962	4811	NID1	nidogen 1 [Source:HGNC Symbol;Acc:HGNC:7821]
ENSG00000141639	5596	MAPK4	mitogen-activated protein kinase 4 [Source:HGNC Symbol;Acc:HGNC:6878]
ENSG00000124159	8785	MATN4	matrilin 4 [Source:HGNC Symbol;Acc:HGNC:6910]
ENSG00000100906	4792	NFKBIA	nuclear factor of kappa light polypeptide gene enhancer in B-cells inhibitor, alpha [Source:HGNC Symbol;Acc:HGNC:7797]
ENSG00000072062	5566	PRKACA	protein kinase, cAMP-dependent, catalytic, alpha [Source:HGNC Symbol;Acc:HGNC:9380]

ENSG00000120910	5533	PPP3CC	protein phosphatase 3, catalytic subunit, gamma isozyme [Source:HGNC Symbol;Acc:HGNC:9316]
ENSG00000106348	3614	IMPDH1	IMP (inosine 5'-monophosphate) dehydrogenase 1 [Source:HGNC Symbol;Acc:HGNC:6052]
ENSG00000122707	8434	RECK	reversion-inducing-cysteine-rich protein with kazal motifs [Source:HGNC Symbol;Acc:HGNC:11345]
ENSG00000169245	3627	CXCL10	chemokine (C-X-C motif) ligand 10 [Source:HGNC Symbol;Acc:HGNC:10637]
ENSG00000116147	7143	TNR	tenascin R [Source:HGNC Symbol;Acc:HGNC:11953]
ENSG00000159248	57369	GJD2	gap junction protein, delta 2, 36kDa [Source:HGNC Symbol;Acc:HGNC:19154]
ENSG00000170417	130827	TMEM182	transmembrane protein 182 [Source:HGNC Symbol;Acc:HGNC:26391]
ENSG00000071051	8440	NCK2	NCK adaptor protein 2 [Source:HGNC Symbol;Acc:HGNC:7665]
ENSG00000158125	7498	XDH	xanthine dehydrogenase [Source:HGNC Symbol;Acc:HGNC:12805]
ENSG00000101421	128866	CHMP4B	charged multivesicular body protein 4B [Source:HGNC Symbol;Acc:HGNC:16171]
ENSG00000124145	6385	SDC4	syndecan 4 [Source:HGNC Symbol;Acc:HGNC:10661]
ENSG00000181019	1728	NQO1	NAD(P)H dehydrogenase, quinone 1 [Source:HGNC Symbol;Acc:HGNC:2874]
ENSG00000080815	5663	PSEN1	presenilin 1 [Source:HGNC Symbol;Acc:HGNC:9508]
ENSG00000171100	4534	MTM1	myotubularin 1 [Source:HGNC Symbol;Acc:HGNC:7448]
ENSG00000162736	23385	NCSTN	nicastrin [Source:HGNC Symbol;Acc:HGNC:17091]
ENSG00000065559	6416	MAP2K4	mitogen-activated protein kinase kinase 4 [Source:HGNC Symbol;Acc:HGNC:6844]
ENSG00000198836	4976	OPA1	optic atrophy 1 (autosomal dominant) [Source:HGNC Symbol;Acc:HGNC:8140]
ENSG00000197442	4217	MAP3K5	mitogen-activated protein kinase kinase kinase 5 [Source:HGNC Symbol;Acc:HGNC:6857]
ENSG00000008441	4784	NFIX	nuclear factor I/X (CCAAT-binding transcription factor) [Source:HGNC Symbol;Acc:HGNC:7788]
ENSG00000107859	5309	PITX3	paired-like homeodomain 3 [Source:HGNC Symbol;Acc:HGNC:9006]
ENSG00000112619	5961	PRPH2	peripherin 2 (retinal degeneration, slow) [Source:HGNC Symbol;Acc:HGNC:9942]
ENSG00000112096	6648	SOD2	superoxide dismutase 2, mitochondrial [Source:HGNC Symbol;Acc:HGNC:11180]

ENSG00000184500	5627	PROS1	protein S (alpha) [Source:HGNC Symbol;Acc:HGNC:9456]
ENSG00000074527	59277	NTN4	netrin 4 [Source:HGNC Symbol;Acc:HGNC:13658]
ENSG00000282110	5596	MAPK4	mitogen-activated protein kinase 4 [Source:HGNC Symbol;Acc:HGNC:6878]
ENSG00000102882	5595	MAPK3	mitogen-activated protein kinase 3 [Source:HGNC Symbol;Acc:HGNC:6877]
ENSG00000171223	3726	JUNB	jun B proto-oncogene [Source:HGNC Symbol;Acc:HGNC:6205]
ENSG00000177606	3725	JUN	jun proto-oncogene [Source:HGNC Symbol;Acc:HGNC:6204]
ENSG00000223919	4049	LTA	lymphotoxin alpha [Source:HGNC Symbol;Acc:HGNC:6709]
ENSG00000167748	3816	KLK1	kallikrein 1 [Source:HGNC Symbol;Acc:HGNC:6357]
ENSG00000008394	4257	MGST1	microsomal glutathione S-transferase 1 [Source:HGNC Symbol;Acc:HGNC:7061]
ENSG00000129038	4016	LOXL1	lysyl oxidase-like 1 [Source:HGNC Symbol;Acc:HGNC:6665]
ENSG00000003436	7035	TFPI	tissue factor pathway inhibitor (lipoprotein-associated coagulation inhibitor) [Source:HGNC Symbol;Acc:HGNC:11760]
ENSG00000228849	7124	TNF	tumor necrosis factor [Source:HGNC Symbol;Acc:HGNC:11892]
ENSG00000137745	4322	MMP13	matrix metallopeptidase 13 [Source:HGNC Symbol;Acc:HGNC:7159]
ENSG00000100122	1414	CRYBB1	crystallin, beta B1 [Source:HGNC Symbol;Acc:HGNC:2397]
ENSG00000145414	92345	NAF1	nuclear assembly factor 1 ribonucleoprotein [Source:HGNC Symbol;Acc:HGNC:25126]
ENSG00000116785	10878	CFHR3	complement factor H-related 3 [Source:HGNC Symbol;Acc:HGNC:16980]
ENSG00000108255	1411	CRYBA1	crystallin, beta A1 [Source:HGNC Symbol;Acc:HGNC:2394]
ENSG00000067182	7132	TNFRSF1A	tumor necrosis factor receptor superfamily, member 1A [Source:HGNC Symbol;Acc:HGNC:11916]
ENSG00000118231	1421	CRYGD	crystallin, gamma D [Source:HGNC Symbol;Acc:HGNC:2411]
ENSG00000135919	5270	SERPINE2	serpin peptidase inhibitor, clade E (nexin, plasminogen activator inhibitor type 1), member 2 [Source:HGNC Symbol;Acc:HGNC:8951]
ENSG00000174562	55554	KLK15	kallikrein-related peptidase 15 [Source:HGNC Symbol;Acc:HGNC:20453]
ENSG00000163254	1420	CRYGC	crystallin, gamma C [Source:HGNC Symbol;Acc:HGNC:2410]
ENSG00000166484	5598	MAPK7	mitogen-activated protein kinase 7 [Source:HGNC Symbol;Acc:HGNC:6880]

ENSG00000000971	3075	CFH	complement factor H [Source:HGNC Symbol;Acc:HGNC:4883]
ENSG00000204983	5644	PRSS1	protease, serine, 1 (trypsin 1) [Source:HGNC Symbol;Acc:HGNC:9475]
ENSG00000196431	1413	CRYBA4	crystallin, beta A4 [Source:HGNC Symbol;Acc:HGNC:2396]
ENSG00000167749	9622	KLK4	kallikrein-related peptidase 4 [Source:HGNC Symbol;Acc:HGNC:6365]
ENSG00000169439	6383	SDC2	syndecan 2 [Source:HGNC Symbol;Acc:HGNC:10659]
ENSG00000196878	3914	LAMB3	laminin, beta 3 [Source:HGNC Symbol;Acc:HGNC:6490]
ENSG00000132693	1401	CRP	C-reactive protein, pentraxin-related [Source:HGNC Symbol;Acc:HGNC:2367]
ENSG00000182187	1419	CRYGB	crystallin, gamma B [Source:HGNC Symbol;Acc:HGNC:2409]
ENSG00000135862	3915	LAMC1	laminin, gamma 1 (formerly LAMB2) [Source:HGNC Symbol;Acc:HGNC:6492]
ENSG00000225614	84627	ZNF469	zinc finger protein 469 [Source:HGNC Symbol;Acc:HGNC:23216]
ENSG00000157227	4323	MMP14	matrix metallopeptidase 14 (membrane-inserted) [Source:HGNC Symbol;Acc:HGNC:7160]
ENSG00000127191	7186	TRAF2	TNF receptor-associated factor 2 [Source:HGNC Symbol;Acc:HGNC:12032]
ENSG00000183943	5613	PRKX	protein kinase, X-linked [Source:HGNC Symbol;Acc:HGNC:9441]
ENSG00000177663	23765	IL17RA	interleukin 17 receptor A [Source:HGNC Symbol;Acc:HGNC:5985]
ENSG00000099625	255057	C19orf26	chromosome 19 open reading frame 26 [Source:HGNC Symbol;Acc:HGNC:28617]
ENSG00000134013	4017	LOXL2	lysyl oxidase-like 2 [Source:HGNC Symbol;Acc:HGNC:6666]
ENSG00000259207	3690	ITGB3	integrin, beta 3 (platelet glycoprotein IIIa, antigen CD61) [Source:HGNC Symbol;Acc:HGNC:6156]
ENSG00000163820	79443	FYCO1	FYVE and coiled-coil domain containing 1 [Source:HGNC Symbol;Acc:HGNC:14673]
ENSG00000095464	5146	PDE6C	phosphodiesterase 6C, cGMP-specific, cone, alpha prime [Source:HGNC Symbol;Acc:HGNC:8787]
ENSG00000154229	5578	PRKCA	protein kinase C, alpha [Source:HGNC Symbol;Acc:HGNC:9393]
ENSG00000035862	7077	TIMP2	TIMP metallopeptidase inhibitor 2 [Source:HGNC Symbol;Acc:HGNC:11821]
ENSG00000053747	3909	LAMA3	laminin, alpha 3 [Source:HGNC Symbol;Acc:HGNC:6483]
ENSG00000170949	90338	ZNF160	zinc finger protein 160 [Source:HGNC Symbol;Acc:HGNC:12948]
ENSG00000244752	1415	CRYBB2	crystallin, beta B2 [Source:HGNC Symbol;Acc:HGNC:2398]

ENSG00000122591	84668	FAM126A	family with sequence similarity 126, member A [Source:HGNC Symbol;Acc:HGNC:24587]
ENSG00000122729	48	ACO1	aconitase 1, soluble [Source:HGNC Symbol;Acc:HGNC:117]
ENSG00000064601	5476	CTSA	cathepsin A [Source:HGNC Symbol;Acc:HGNC:9251]
ENSG00000117450	5052	PRDX1	peroxiredoxin 1 [Source:HGNC Symbol;Acc:HGNC:9352]
ENSG00000121879	5290	PIK3CA	phosphatidylinositol-4,5-bisphosphate 3-kinase, catalytic subunit alpha [Source:HGNC Symbol;Acc:HGNC:8975]
ENSG00000109846	1410	CRYAB	crystallin, alpha B [Source:HGNC Symbol;Acc:HGNC:2389]
ENSG00000142515	354	KLK3	kallikrein-related peptidase 3 [Source:HGNC Symbol;Acc:HGNC:6364]
ENSG00000129451	5655	KLK10	kallikrein-related peptidase 10 [Source:HGNC Symbol;Acc:HGNC:6358]
ENSG00000105329	7040	TGFB1	transforming growth factor, beta 1 [Source:HGNC Symbol;Acc:HGNC:11766]
ENSG00000005249	5577	PRKAR2B	protein kinase, cAMP-dependent, regulatory, type II, beta [Source:HGNC Symbol;Acc:HGNC:9392]
ENSG00000060069	9150	CTDP1	CTD (carboxy-terminal domain, RNA polymerase II, polypeptide A) phosphatase, subunit 1 [Source:HGNC Symbol;Acc:HGNC:2498]
ENSG00000145675	5295	PIK3R1	phosphoinositide-3-kinase, regulatory subunit 1 (alpha) [Source:HGNC Symbol;Acc:HGNC:8979]
ENSG00000012660	60481	ELOVL5	ELOVL fatty acid elongase 5 [Source:HGNC Symbol;Acc:HGNC:21308]
ENSG00000160211	2539	G6PD	glucose-6-phosphate dehydrogenase [Source:HGNC Symbol;Acc:HGNC:4057]
ENSG00000175104	7189	TRAF6	TNF receptor-associated factor 6, E3 ubiquitin protein ligase [Source:HGNC Symbol;Acc:HGNC:12036]
ENSG00000178209	5339	PLEC	plectin [Source:HGNC Symbol;Acc:HGNC:9069]
ENSG00000050555	10319	LAMC3	laminin, gamma 3 [Source:HGNC Symbol;Acc:HGNC:6494]
ENSG00000198400	4914	NTRK1	neurotrophic tyrosine kinase, receptor, type 1 [Source:HGNC Symbol;Acc:HGNC:8031]
ENSG00000106333	5118	PCOLCE	procollagen C-endopeptidase enhancer [Source:HGNC Symbol;Acc:HGNC:8738]
ENSG00000167751	3817	KLK2	kallikrein-related peptidase 2 [Source:HGNC Symbol;Acc:HGNC:6363]
ENSG00000186340	7058	THBS2	thrombospondin 2 [Source:HGNC Symbol;Acc:HGNC:11786]

ENSG00000106397	8985	PLOD3	procollagen-lysine, 2-oxoglutarate 5-dioxygenase 3 [Source:HGNC Symbol;Acc:HGNC:9083]
ENSG00000130702	3911	LAMA5	laminin, alpha 5 [Source:HGNC Symbol;Acc:HGNC:6485]
ENSG00000006062	9020	MAP3K14	mitogen-activated protein kinase kinase kinase 14 [Source:HGNC Symbol;Acc:HGNC:6853]
ENSG00000118503	7128	TNFAIP3	tumor necrosis factor, alpha-induced protein 3 [Source:HGNC Symbol;Acc:HGNC:11896]
ENSG00000100030	5594	MAPK1	mitogen-activated protein kinase 1 [Source:HGNC Symbol;Acc:HGNC:6871]
ENSG00000106366	5054	SERPINE1	serpin peptidase inhibitor, clade E (nexin, plasminogen activator inhibitor type 1), member 1 [Source:HGNC Symbol;Acc:HGNC:8583]
ENSG00000164610	6100	RP9	retinitis pigmentosa 9 (autosomal dominant) [Source:HGNC Symbol;Acc:HGNC:10288]
ENSG00000101966	331	XIAP	X-linked inhibitor of apoptosis, E3 ubiquitin protein ligase [Source:HGNC Symbol;Acc:HGNC:592]
ENSG00000179774	220202	ATOH7	atonal homolog 7 (Drosophila) [Source:HGNC Symbol;Acc:HGNC:13907]
ENSG00000197461	5154	PDGFA	platelet-derived growth factor alpha polypeptide [Source:HGNC Symbol;Acc:HGNC:8799]
ENSG00000102076	5956	OPN1LW	opsin 1 (cone pigments), long-wave-sensitive [Source:HGNC Symbol;Acc:HGNC:9936]
ENSG00000204103	9935	MAFB	v-maf avian musculoaponeurotic fibrosarcoma oncogene homolog B [Source:HGNC Symbol;Acc:HGNC:6408]
ENSG00000268221	2652	OPN1MW	opsin 1 (cone pigments), medium-wave-sensitive [Source:HGNC Symbol;Acc:HGNC:4206]
ENSG00000132386	5176	SERPINF1	serpin peptidase inhibitor, clade F (alpha-2 antiplasmin, pigment epithelium derived factor), member 1 [Source:HGNC Symbol;Acc:HGNC:8824]
ENSG00000172936	4615	MYD88	myeloid differentiation primary response 88 [Source:HGNC Symbol;Acc:HGNC:7562]
ENSG00000173976	84839	RAX2	retina and anterior neural fold homeobox 2 [Source:HGNC Symbol;Acc:HGNC:18286]
ENSG00000125966	10893	MMP24	matrix metallopeptidase 24 (membrane-inserted) [Source:HGNC Symbol;Acc:HGNC:7172]
ENSG00000043355	7546	ZIC2	Zic family member 2 [Source:HGNC Symbol;Acc:HGNC:12873]

ENSG00000100503	51199	NIN	ninein (GSK3B interacting protein) [Source:HGNC Symbol;Acc:HGNC:14906]
ENSG00000163914	6010	RHO	rhodopsin [Source:HGNC Symbol;Acc:HGNC:10012]
ENSG00000125850	58495	OVOL2	ovo-like zinc finger 2 [Source:HGNC Symbol;Acc:HGNC:15804]
ENSG00000138061	1545	CYP1B1	cytochrome P450, family 1, subfamily B, polypeptide 1 [Source:HGNC Symbol;Acc:HGNC:2597]
ENSG00000011422	5329	PLAUR	plasminogen activator, urokinase receptor [Source:HGNC Symbol;Acc:HGNC:9053]
ENSG00000100058	1416	CRYBB2P1	crystallin, beta B2 pseudogene 1 [Source:HGNC Symbol;Acc:HGNC:2399]
ENSG00000145901	10318	TNIP1	TNFAIP3 interacting protein 1 [Source:HGNC Symbol;Acc:HGNC:16903]
ENSG00000275365	4320	MMP11	matrix metallopeptidase 11 [Source:HGNC Symbol;Acc:HGNC:7157]
ENSG00000123700	3759	KCNJ2	potassium channel, inwardly rectifying subfamily J, member 2 [Source:HGNC Symbol;Acc:HGNC:6263]
ENSG00000196455	30849	PIK3R4	phosphoinositide-3-kinase, regulatory subunit 4 [Source:HGNC Symbol;Acc:HGNC:8982]
ENSG00000091622	83394	PITPNM3	PITPNM family member 3 [Source:HGNC Symbol;Acc:HGNC:21043]
ENSG00000058085	3918	LAMC2	laminin, gamma 2 [Source:HGNC Symbol;Acc:HGNC:6493]
ENSG00000117601	462	SERPINC1	serpin peptidase inhibitor, clade C (antithrombin), member 1 [Source:HGNC Symbol;Acc:HGNC:775]
ENSG00000130287	1463	NCAN	neurocan [Source:HGNC Symbol;Acc:HGNC:2465]
ENSG00000178531	404217	CTXN1	cortexin 1 [Source:HGNC Symbol;Acc:HGNC:31108]
ENSG00000105989	7472	WNT2	wingless-type MMTV integration site family member 2 [Source:HGNC Symbol;Acc:HGNC:12780]
ENSG00000278843	79148	MMP28	matrix metallopeptidase 28 [Source:HGNC Symbol;Acc:HGNC:14366]
ENSG00000274474	7531	YWHAE	tyrosine 3-monooxygenase/tryptophan 5-monooxygenase activation protein, epsilon [Source:HGNC Symbol;Acc:HGNC:12851]
ENSG00000281877	6195	RPS6KA1	ribosomal protein S6 kinase, 90kDa, polypeptide 1 [Source:HGNC Symbol;Acc:HGNC:10430]
ENSG00000280495	51399	TRAPPC4	trafficking protein particle complex 4 [Source:HGNC Symbol;Acc:HGNC:19943]
ENSG00000281897	6942	TCF20	transcription factor 20 (AR1) [Source:HGNC Symbol;Acc:HGNC:11631]

ENSG00000100448	1511	CTSG	cathepsin G [Source:HGNC Symbol;Acc:HGNC:2532]
ENSG00000109072	7448	VTN	vitronectin [Source:HGNC Symbol;Acc:HGNC:12724]
ENSG00000142173	1292	COL6A2	collagen, type VI, alpha 2 [Source:HGNC Symbol;Acc:HGNC:2212]
ENSG00000115718	5624	PROC	protein C (inactivator of coagulation factors Va and VIIIa) [Source:HGNC Symbol;Acc:HGNC:9451]
ENSG00000181449	6657	SOX2	SRY (sex determining region Y)-box 2 [Source:HGNC Symbol;Acc:HGNC:11195]
ENSG00000075891	5076	PAX2	paired box 2 [Source:HGNC Symbol;Acc:HGNC:8616]
ENSG00000176692	2303	FOXC2	forkhead box C2 [Source:HGNC Symbol;Acc:HGNC:3801]
ENSG00000183770	668	FOXL2	forkhead box L2 [Source:HGNC Symbol;Acc:HGNC:1092]
ENSG00000165462	401	ARIX(=PHO	paired-like homeobox 2a [Source:HGNC Symbol;Acc:HGNC:691]
ENSG00000137203	7020	TFAP2A	transcription factor AP-2 alpha (activating enhancer binding protein 2 alpha) [Source:HGNC Symbol;Acc:HGNC:11742]
ENSG00000183010	5831	PYCR1	pyrroline-5-carboxylate reductase 1 [Source:HGNC Symbol;Acc:HGNC:9721]
ENSG00000196924	2316	FLNA	filamin A, alpha [Source:HGNC Symbol;Acc:HGNC:3754]
ENSG00000134595	6658	SOX3	SRY (sex determining region Y)-box 3 [Source:HGNC Symbol;Acc:HGNC:11199]
ENSG00000048052	9734	HDAC9	histone deacetylase 9 [Source:HGNC Symbol;Acc:HGNC:14065]
ENSG00000124479	4693	NDP	Norrie disease (pseudoglioma) [Source:HGNC Symbol;Acc:HGNC:7678]
ENSG00000185960	6473	SHOX	short stature homeobox [Source:HGNC Symbol;Acc:HGNC:10853]
ENSG00000187140	27022	FOXD3	forkhead box D3 [Source:HGNC Symbol;Acc:HGNC:3804]
ENSG00000120149	4488	MSX2	msh homeobox 2 [Source:HGNC Symbol;Acc:HGNC:7392]
ENSG00000186790	2301	FOXE3	forkhead box E3 [Source:HGNC Symbol;Acc:HGNC:3808]
ENSG00000130508	7837	PXDN	peroxidasin [Source:HGNC Symbol;Acc:HGNC:14966]
ENSG00000187676	145173	B3GLCT	beta 3-glucosyltransferase [Source:HGNC Symbol;Acc:HGNC:20207]
