# Supplementary material for: Novel Mutations Associated With Various Types of Corneal Dystrophies in a Han Chinese Population
Source: Front Genet. 2019 Aug 29;10:881. doi: 10.3389/fgene.2019.00881 (PMC6726741; doi:10.3389/fgene.2019.00881)
Supplement: Supplementary file 3 [file DataSheet_2.pdf]

Table S2. Primers used for validation by Sanger sequencing

| Gene | Variant | Forward Primer | Reverse Primer |
|---|---|---|---|
| *TGFBI* | R124H/L/C | 5'-AGCTGTGCTCTGCTGTGTTTA-3' | 5'-AGGCAGTTCCCCATAAGAGT-3' |
| | F540S/R555W | 5'-GCATACGGAGGGCATGAAAAC-3' | 5'-CTTGCATGGTTCTGACAGTGG-3' |
| | L565H | 5'-ACTGCTGGGGCAGATTTGTG-3' | 5'-CCAGCCTTTGATTTGCAGGAC-3' |
| *CHST6* | S51X/F55S | 5'-CAAACACGTCCATGTCGCAC-3' | 5'-TTCATGGGATGCTCGGGTCT-3' |
| | exon3 | 5'-TCCACTAATTTCGGGGGTGC-3' | 5'-TGGGCCAACTCTTCAACCAG-3' |
| *SLC4A11* | G412R | 5'-GTCCATGCGTAGAAGGAGTTGA-3' | 5'-CACTGATGGTACGTGGCCTCT-3' |
| | Q676R/L732fs | 5'-TTCACGTTCACAATCCTGCG-3' | 5'-ATAGCAGTAGCCTGTCCCCT-3' |
| | R755W | 5'-TAGAGGAAGAGGCCATAGAGCAC-3' | 5'-CATGGGACATAGGGAGGAGTGAG-3' |
| | R869H | 5'-GATGAGCCTGGGTCAGAGAGAA-3' | 5'-GGAAGATCCACTACTTCACGGG-3' |
| *AGBL1* | R748H | 5'-GTCTTGCAGACCACAGAGGAA-3' | 5'-GAGTACCGAGGCCTCTCCAA-3' |
| | R1028X | 5'-TGAAAAATGCCTGTGTGTAGAAGTC-3' | 5'-GCAGCATCCTCTTTTTCATCAGTAG-3' |
| *COL17A1* | P1185L | 5'-CCACAGTGCCCTACTATGTAAGTAA-3' | 5'-CACCATCTCTTTTCTTTCTTGGACC-3' |
